# Effect of malocclusion on jaw motor function and chewing in children: a systematic review

**DOI:** 10.1007/s00784-021-04356-y

**Published:** 2022-01-05

**Authors:** Abdulrahman Alshammari, Nabeel Almotairy, Abhishek Kumar, Anastasios Grigoriadis

**Affiliations:** 1 Department of Orthodontics, College of Dentistry, University of Hail, Hail, Saudi Arabia; 2 Department of Orthodontics and Pediatric Dentistry, College of Dentistry, Qassim University, Buraidah, Saudi Arabia; 3 Section of Oral Rehabilitation, Department of Dental Medicine, Karolinska Institutet, Alfred Nobels Allé 8, Box 4064, 141 04 Huddinge, Sweden

**Keywords:** Mastication, Electromyography, Bite force, Masticatory muscles, Jaw kinematics, Early orthodontics, Chewing performance

## Abstract

**Objective:**

To investigate the effects of dental/skeletal malocclusion and orthodontic treatment on four main objective parameters of chewing and jaw function (maximum occlusal bite force [MOBF], masticatory muscle electromyography [EMG], jaw kinematics, and chewing efficiency/performance) in healthy children.

**Materials and methods:**

Systematic searches were conducted in MEDLINE (OVID), Embase, and the Web of Science Core Collection. Studies that examined the four parameters in healthy children with malocclusions were included. The quality of studies and overall evidence were assessed using the Joanna Briggs Institute and GRADE tools, respectively.

**Results:**

The searches identified 8192 studies; 57 were finally included. The quality of included studies was high in nine studies, moderate in twenty-three studies, and low in twenty-five studies. During the primary dentition, children with malocclusions showed similar MOBF and lower chewing efficiency compared to control subjects. During mixed/permanent dentition, children with malocclusion showed lower MOBF and EMG activity and chewing efficiency compared to control subjects. The jaw kinematics of children with unilateral posterior crossbite showed a larger jaw opening angle and a higher frequency of reverse chewing cycles compared to crossbite-free children. There was a low to moderate level of evidence on the effects of orthodontic treatment in restoring normal jaw function.

**Conclusions:**

Based on the limitations of the studies included, it is not entirely possible to either support or deny the influence of dental/skeletal malocclusion traits on MOBF, EMG, jaw kinematics, and masticatory performance in healthy children. Furthermore, well-designed longitudinal studies may be needed to determine whether orthodontic treatments can improve chewing function in general.

**Clinical relevance:**

Comprehensive orthodontic treatment, which includes evaluation and restoration of function, may or may not mitigate the effects of malocclusion and restore normal chewing function.

**Supplementary Information:**

The online version contains supplementary material available at 10.1007/s00784-021-04356-y.

## Introduction

Mastication is a complex sensory-motor interaction between the central nervous system and the peripheral masticatory apparatus. The semi-automatic, rhythmic act of mastication is initiated by the central nervous system and fine-tuned by inputs from receptors embedded in the orofacial area [1]. The process of mastication and the coordination of masticatory movements depend largely on the harmonious interaction between the peripheral inputs and the higher centers of the brain [2–5]. In general, the process of mastication involves the proper placement of food morsels in between the teeth, crushing (the food) into smaller pieces, and mixing them with saliva to form a coherent and swallowable bolus [6, 7]. In addition to the physical comminution of food, mastication plays an integral role in the process of swallowing, salivary secretion [8], taste and flavor perception, digestion, and nutrition [6, 7] in humans. Therefore, it is believed that impaired chewing function can have a cascading effect on overall health and quality of life [9–12].

In children, the sensorimotor systems must adapt to substantial morphological changes during growth and development. The orofacial area is particularly challenged by the growth and development of the jaws and the transition from primary to permanent dentition [13]. Studies on masticatory movements suggest that children have a characteristic chewing pattern that differs from that of adults and that certain movement parameters (jaw opening and closing velocities) change with age [14, 15]. A series of well-controlled studies in children with normal occlusion have shown that chewing and jaw motor skills develop gradually with age [16–18]. In particular, masticatory behavior has been shown to be most prone to deviation from normal in 6-year-old children (deciduous dentition) compared to adults. However, children in the late-mixed to early-permanent dentition stages show similar chewing behavior to adults. As mentioned earlier, the transition from the primary to permanent dentition is a lengthy process that involves an intense transformation of craniofacial form and function. Often, this transformation results in drastic changes in skeletal mass, skeletal shape, muscle mass, and muscle geometry, which confront the nervous system with dynamically varying systems that it must control. In addition, these changes may also be associated with abnormalities of occlusion and jaw function (i.e., malocclusion). Dental and/or skeletal malocclusions are common orofacial dysfunctions in humans that have multifactorial causes, including genetic and environmental factors. It is well-established that dental malocclusions can have negative effects not only on normal jaw development and chewing functions (for review, see [19]) but also on the psychosocial well-being of children [20].

Orthodontic correction is often the preferred treatment method for correcting malocclusions. In more severe cases where orthodontic treatment appears inadequate, orthognathic surgery is recommended. Most orthodontic examinations involve a physical/radiographic assessment of deformities without evaluation of function. Orthodontic treatment is generally aimed at improving dental occlusion and enhancing esthetics, with little attention to restoring or optimizing chewing function. A number of studies have been published describing the effects of mandibular muscle activity in individuals with different categories of malocclusion (for a review: [21]). Although orthodontic corrections result in ideal occlusal restoration and improved esthetics, the question of whether they also improve the chewing function remains to be investigated. Previous studies have shown that people with normal occlusion had better chewing efficiency than those with orthodontically treated or untreated malocclusion [22]. However, it is unclear whether the presence of malocclusion in healthy children affects normal chewing and jaw motor function.

In a previous systematic review, we examined the development of common objective indicators of chewing and jaw function (i.e., maximum occlusal bite force [MOBF], electromyography [EMG], jaw kinematics, and chewing efficiency) in healthy children without malocclusion [23]. Building on the previous work, the present systematic review aims to investigate the influence of dental/skeletal malocclusion on the development of the above-mentioned objective indicators of chewing and jaw function in healthy children. One of the general goals of orthodontic treatment is to restore jaw function by establishing a normal, stable, and harmonious relationship between dental and skeletal structures. Therefore, the current study also investigates whether orthodontic treatment contributes to the restoration of the above-mentioned parameters.

## Material and methods

The protocol of the current systematic review was pre-registered in the Open Science Framework repository (https://osf.io/7k3ue/) and was conducted according to the recent updates of the PRISMA-P guidelines [24].

### Information sources and search strategy

Systematic searches were conducted in MEDLINE (Ovid), Embase, and Web of Science Core Collection databases from inception until October 29, 2021. The search strategy (Supplementary file 1) was created using MeSH/Emtree terms with relevant free text terms, and truncated and/or combined with proximity operators, where appropriate. There were no search restrictions on date or type of publication, but only studies published in English were included. The database search was supplemented by a manual search of gray literature and Google Scholar using free text terms such as “chewing in children,” “bite force,” “electromyography,” and “jaw kinematics.” In addition, the reference lists of included studies were searched for any potentially eligible studies.

### The screening and selection of eligible studies

Duplicate studies were automatically removed from the search results using EndNote reference management software and were confirmed by a manual inspection. All shortlisted studies were then exported to a Microsoft Excel spreadsheet, where two independent authors (AA and NA) screened the titles/abstracts of the study list. Clinical studies that investigated the objective parameters of jaw motor function and mastication in children with malocclusion, with or without cross-comparison to the malocclusion-free group of children and/or adults, were included. However, studies on children with other orofacial anomalies or in a language other than English were excluded. All reviews (systematic or narrative), study protocols, letters to editor, opinion articles, commentaries, and case reports were also excluded. Based on the inclusion/exclusion criteria, studies were categorized as “included,” “excluded,” or “undecided” using a predefined scheme. Indecision about the inclusion of a study was resolved by joint discussion and/or consultation with a third author (AK), if necessary. The full texts of all included studies were obtained and carefully read by the two independent authors. The studies that met the inclusion/exclusion criteria were finally included in this systematic review. A clear reason was provided for the exclusion of a study, and disagreements about the inclusion/exclusion of a study were again resolved by joint discussion and/or consultation with a third author (if necessary).

### Quality assessment of included studies

The quality of evidence and risk of bias of the included studies were assessed using the Joanna Briggs Institute Critical Appraisal Tool. The purpose of this appraisal is to assess the methodological quality of individual studies and to determine the extent to which a particular study has addressed the possibility of bias in its design, conduct, and analysis. Two separate instruments related to methodological design (i.e., cross-sectional or prospective cohorts studies) were used [25]. The instrument/tool consists of several methodological questions on the representativeness of the study sample and inclusion criteria, the study setting and design, the validity and reliability of the exposure and outcomes, and the adequacy of the statistical analysis. A cumulative score was calculated for each study by adding the positive responses of the instrument questions. Each study was then classified as high quality (score between 100 and 80), moderate quality (score between 79 and 60), or low quality (score below 60) based on its cumulative score. Note that no study was excluded based on its quality score. Data extracted from the included studies encompassed demographics of study sample, type of malocclusion, orthodontic treatment/retention (if any), objective parameter for mastication measured, main outcomes, and study quality score. In addition, the Grading of Recommendations Assessment, Development, and Evaluation (GRADE) tool [26] was used to assess the overall quality of the evidence of the impact/effect of orthodontic treatment on the selected parameters of jaw motor function and mastication.

## Results

**Fig. 1:**
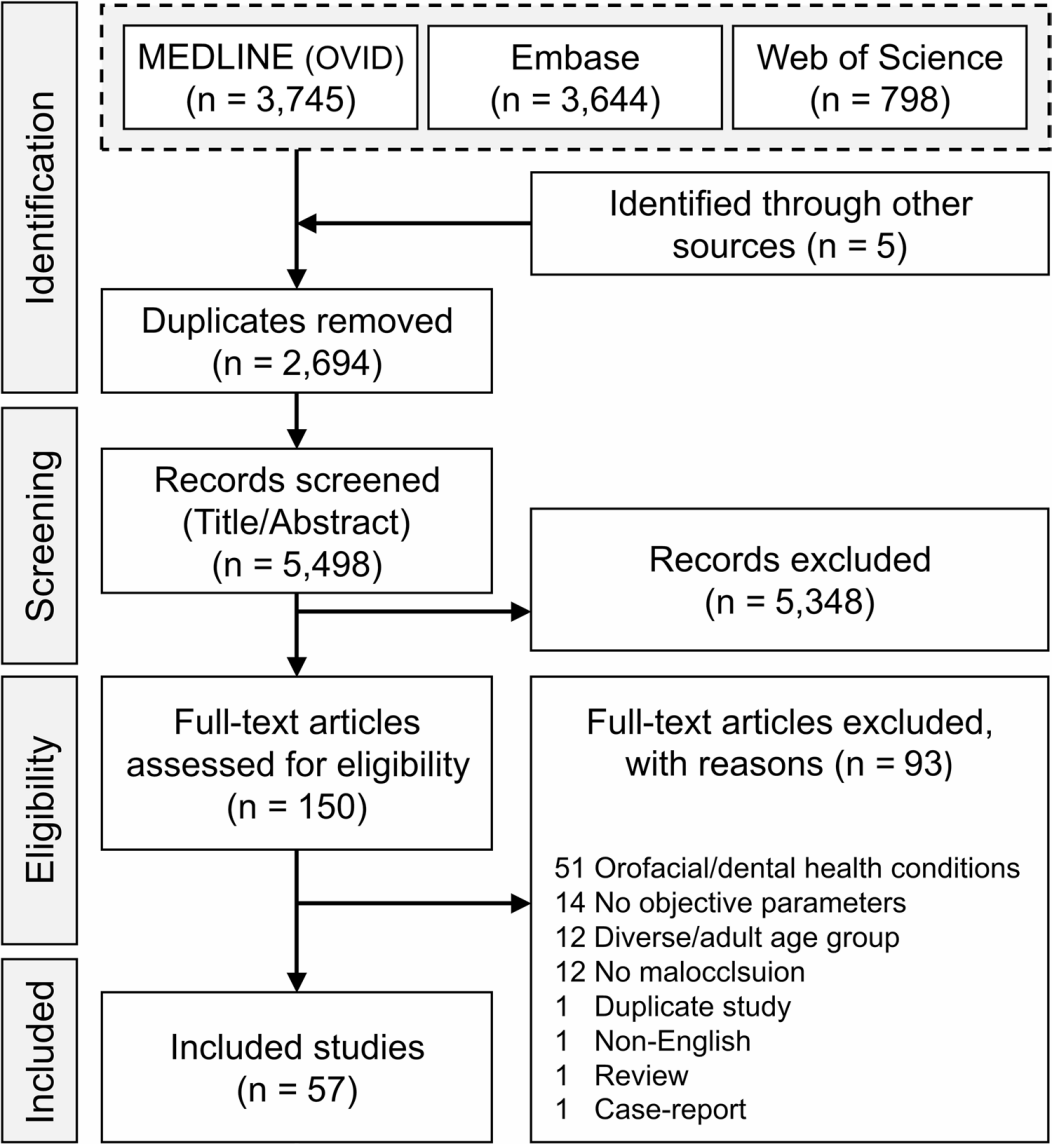
PRISMA flow chart showing the database search result and the selection process of eligible studies

The database search resulted in 8192 studies, with five studies identified by manual search. After the removal of duplicates and screening of the titles/abstracts of the search results, a list of 150 potentially eligible studies remained for inclusion. Full-text review of the potentially eligible studies resulted in a final list of 57 included studies (Fig. 1).

The quality of the included studies was high in nine studies [27–35], moderate in twenty-three studies [36–58], and low in twenty-five studies [22, 59–82]. The detailed characteristics and the quality outcome of the included studies are shown in Table 1 and Supplementary file 2. The majority of the included studies were cross-sectional, while twenty-five studies were prospective cohorts [22, 29, 31, 34, 38, 40, 42, 47, 49–52, 54, 55, 57, 58, 61, 67, 69, 71, 73, 76, 79–81]. All the included studies investigated the four selected parameters for jaw function and chewing on various malocclusion types with/without cross-comparison to malocclusion-free group (control group). Among the included studies, eight studies investigated more than one chewing parameter [33, 38, 48, 55, 63, 65, 67, 70]. Table 2 presents the pooled results of the four objective parameters.

**Table 1:** Characteristics of the included studies

Study	Malocclusion group N; gender; age	Malocclusion type(s)	Orthodontic treatment	Orthodontic retention	Control group 3 N; gender; age	Method(s)	Quality outcome
Ahlgren (1967)	290; 9 F:21 M; 8–16 yr	Cl I, II/1, II/2 and III AOB, deep bite & Ant & post XB	NA	NA	30; 156 F:134 M; 8–16 yr	Jaw kinematics	Low
Ahlgren et al., (1973)	15; ?; 9–13 yr	Cl II/1	NA	NA	15; ?; 9–11 yr	1- EMG 2- Jaw kinematics	Low
Alarcón et al., (2000)	30; 17 F:13 M; 12.2 yr	UPXB	NA	NA	30; 16 F:14 M; 12.5 yr	EMG	Low
Alarón et al., (2009)	30; 15 F:15 M, 10–12 yr	UPXB	NA	NA	30; 15 F:15 M; 10–12 yr	EMG	Moderate
Andrade et al., (2009)	20; 8 F:12 M; 7–10 yr	UPXB	NA	NA	16; 6 F:10 M; 7–10 yr	EMG	Moderate
Andrade et al., (2010)	17; ?; 7–10 yr	UPXB	NA	NA	17; ?; 7–10 yr	1- EMG 2- Jaw kinematics	Low
Antonarakis & Kiliaridis (2015)	20; 6 F:14 M; 9–13 yr	Cl II/1	Activator (1 yr)	NA	20; 6 F:14 M; 9–13 yr	MOBF	High
Atonarakis et al., (2012)	25; 8 F:17 M: 9.3–13 yr	Cl II/1	Activator (1–2 yr)	NA	NA	MOBF	Moderate
Atonarakis et al., (2013)	28; 12 F:16 M: 8.5–14.2 yr	Cl II/1	Activator (1–2 yr)	1 yr	NA	MOBF	Moderate
Barrera et al., (2011)	450; 206 F:244 M; 6–15 yr	Cl I and Cl II	NA	NA	NA	Chewing efficiency	High
Ben-Bassat et al. (1993)	36; 19 F:17 M; ?	UPXB	Expansion plate	6 mo	Pre: 29; 17 F:12 M; ? Post: 10; 5 F:5 M; ?	Jaw kinematics	Moderate
Castelo et al., (2007)	21; ?; 3.5–7 yr	UPXB	NA	NA	28; ?; 3.5–7 yr	MOBF	Moderate
Castelo et al., (2010)	35; 20 F:15 M; 3.5–7 yr	UPXB	NA	NA	32; 11 F:21 M; 3.5–7 yr	MOBF	High
Ciavarella et al., (2012)	15; 9 F:6 M; 9–14 yr	UPXB	NA	NA	15; 7 F:8 M; 9–14 yr	EMG	Low
Ciccone de Faria et al., (2010)	30; 20 F:10 M; 6–11 yr	15; Skeletal AOB 15; Dentoalveolar AOB	NA	NA	15; 11 F:4 M; 6–11 yr	EMG	Low
Corrêa et al.,(2018)	51; ?; 7–11 yr	AOB	NA	NA	55; ?; 7–11 yr	Chewing efficiency	Low
Cost et al. 2020	8; 4 F:4 M; 12–19 yr	AOB	NA	NA	8; 4 F:4 M; 12–19 yr	Chewing efficiency	Low
Di Palma et al., (2017)	10; 5 F:5 M; 9–13 yr	Cl II/1	Sander appliance (1 yr)	NA	NA	EMG	Low
Ferrario et al., (1999)	10; 6 F:4 M;16–18 yr	UPXB	NA	NA	20; 10 F:10 M; 16–18 yr	EMG	Moderate
Galbiati et al., (2016)	71; 35 F:36 M; 6–10 yr	UPXB	Hyrax RME	6 mo	NA	1- EMG 2- Jaw kinematics	Low
Gavião et al., (2001)	20; ?; 3–5.5 yr	10; UPXB 10; AOB	NA	NA	10; ?; 3–5.5 yr	Chewing efficiency	Moderate
Go (1981)	12; 2 F:10 M; 10.9 yr	AXB	?	29 mo	5; 3 F:2 M; 12.5 yr 12; 5 F:7 M; 19.8 yr	EMG	Low
Henrikson et al., (1998)	123; only F; 11–15 yr	Cl II	NA	NA	60; only F; 11–15 yr	Chewing efficiency	Moderate
Henrikson et al., (2009)	Tx group: 65; only F; 12.8 ± 1.1 yr No tx group: 58; only F; 12.9 ± 1.0 yr	Cl II	FA	NA	60; only F; 12.7 ± 0.7 yr	Chewing efficiency	Low
Hinotume et al., (1994)	40; 26 F:14 M; 6–12 yr	Teeth crowding	NA	NA	40; 21 F:19 M; 5–12 yr	EMG	Low
Hong et al., (2021)	18; 16 F:2 M; 12–18 yr	Skeletal AOB	Myofunctional appliance (3 mo)	NA	NA	EMG	Moderate
Ingervall et al., (1991)	Group 1: 24; 12 F:12 M; 9.2–12.7 yr Group 2: 15; 8 F:7 M; 8.5–13.1 yr 3Group 3: 16; 10 F:6 M; 9.1–15.4 yr	Group 1 & 2: Cl II/1 Group 3: C I	Group 1: activator without HG Group 2: HG without removable plate Group 3: FA	NA	NA	EMG	Low
Keeling et al., (1991)	9; 2 F:7 M; 115.7 ± 29.5 mo	Functional UPXB	QH (9 patients)	6 to 28 mo	8; 5 F:3 M;106.4 ± 42.2 mo	Jaw kinematics	Moderate
Kecik et al., (2007)	35; 20 F:15 M; 10.6 ± 1.4 yr	Functional UPXB	QH	6 mo	31; 18 F:13 M; 9.8 ± 1.6 yr	EMG	Low
Lenguas et al., (2012)	30; 15 F:15 M, 6–10 yr	Functional UPXB	NA	NA	NA	EMG	Moderate
Lipari et al., (2020)	20; 13 F:7 M; 9.60 ± 1.46 yr	Incompetent lips	NA	NA	20; 10 F:10 M; 9.15 ± 1.66 yr	EMG	High
Lowe and Takada (1984)	55; ?; 11.9 ± 1.8 yr	18; Cl I 25; Cl II/1 12; Cl II/2	NA	NA	NA	EMG	Low
Martín et al., (2012)	25; 15 F:10 M; 10–14 yr	Functional UPXB	QH + FA	1 year	30; 15 F:15 M; 10–14 yr	1- EMG 2- Jaw kinematics	Moderate
Michelotti et al., (2019)	29; 16 F:13 M; 9.6 ± 1.6 yr	Functional UPXB	RME	6 mo	40; 23 F:17 M; 10.5 ± 1.1 yr	EMG	Moderate
Nagata et al., (2002)	10; ?; 3–7 yr	AXB	NA	NA	10; ?; 5–6 yr	Jaw kinematics	Low
Nuño-Licona et al., (1993)	10; ?; 4–10 yr	Cl III	Myofunctional appliance	NA	10; ?; mean age 7.3 yr	EMG	Low
Petrović et al., (2014)	70; ?; 8–12 yr	Cl II/2	Activator	NA	30; ?; 8–12 yr	EMG	Low
Piancino et al., (2012)	Group 1: 26; 16 F:10 M; 10.4 ± 2.7 yr Group 2: 43; 21 F:22 M; 10.2 ± 4.2 yr	Group 1: UAXB Group 2: UPXB	NA	NA	17; 10 F:7 M; 10.6 ± 2.0 yr	Jaw kinematics	Moderate
Piancino et al., (2012)	52; 29 F:23 M; 10.58 ± 1.4 yr	AOB	NA	NA	21; ?; 11.9 ± 0.6 yr	1- EMG 2- Jaw kinematics	High
Piancino et al., (2016)	50; ?; 9.1 ± 2.3 yr	UPXB	“Function generating bite” appliance	5–6 mo	20; ?; 9.5 ± 2.6 yr	1- EMG 2- Jaw kinematics	Moderate
Piancino et al., (2017)	47; ?; 8.3 6 ± 1.1 yr	UPXB	“Function generating bite” appliance	5–6 mo	18; ?; 9.1 ± 0.8 yr	Jaw kinematics	Low
Proffit and Fields (1983)	12; 7 F:5 M; 6–11 yr	Increased lower facial height	NA	NA	18; 9 F:9 M; 6–11 yr	MOBF	Low
Regalo et al., (2018)	64; ?; 6–10 yr	Group 1: grade 2 IOTN- DHC Group 2: grade 3 IOTN- DHC	NA	NA	26, ?, 6–10 yr (Grade 1 IOTN-DHC)	EMG	Low
Rentes et al., (2002)	30; ?; 3–5.5 yr	Crossbite and AOB	NA	NA	30; ?; 3–5.5 yr	MOBF	Moderate
Roldan et al., (2016)	Group 1: 111; 60 F:51 M; 7–15 yr Group 2: 380; 60 F:79 M; 7–15 yr	Group1: Cl I Group 2: Cl II	NA	NA	130; 65 F:65 M; 7–15 yr	MOBF	High
Sabashi et al., (2009)	172; 108 F:64 M; 10–18 yr	41; Cl I 16; Cl II 7; Cl III	NA	NA	NA	EMG	High
Salioni et al., (2005)	16; 9 F:7 M; 6–12.58 yr	Functional UPXB	NA	NA	15; 9 F:6 M; 6–12.75 yr	Jaw kinematics	Moderate
Satygo et al., (2014)	Group 1: 36; ?; 7.6 ± 1.3 yr Group 2: 22; ?; ?	Cl II/1	Group 1: Pre-orthodontic trainer Group 2: No tx	NA	20; ?; 7.6 ± 1.3 yr	EMG	Moderate
Sever et al., (2011)	20; 14 F:6 M; 4.8–5.3 yr	UPXB	NA	NA	10; 5 F:5 M; 5.1–5.3 yr	Jaw kinematics	Moderate
Shiere et al., (1952)	400; ?; 6–15 yr	Cl I, II, III & XP	NA	NA	NA	1- MOBF 2- Chewing efficiency	Low
Sonnesen et al., (2001)	26; 13 F:13 M; 7–13 yr	UPXB	NA	NA	26; 13 F:13 M; 7–13 yr	MOBF	High
Spolaor et al., (2020)	43; ?, 9.5 ± 2.1 yr	Underdeveloped maxilla, UPXB & BPXB	RME	3 mo	10; ?; 9.8 ± 2.2 yr	EMG	Moderate
Takada and Lowe 1985	20; ?; 12.1 ± 1.2 yr	Deep bite + Cl I & II	NA	NA	15; ?; 12.1 ± 1.2 yr	EMG	Low
Throckmorton et al., (2001)	14; ?; 7–11 yr	Functional UPXB	RME	6 mo	14; ?; 7–11 yr	Jaw Kinematics	Moderate
Toro et al. (2006)	355 children to 4 groups: 37; 09 F:28 M; 6 yr3 62; 23 F:39 M; 9 yr 49; 14 F:35 M; 12 yr 50; 12 F:36 M; 15 yr	112; CI I 84; CI II	NA	NA	139 children to 4 groups: 40; 10 F:30 M; 6 yr 37; 15 F:22 M; 9 yr 23; 10 F:13 M; 12 yr 39; 18 F:21 M; 15 yr	Chewing efficiency	High
Yashiro et al., (2004)	11; 6 F:5 M; 10.8 ± 0.11 yr	AXB	5; lingual arches 2; active plates 4; edgewise appliance	2–3 mo	10; 5 F:5 M; 11.2 ± 0.5 yr	Jaw kinematics	Low
Yousefzadeh et al., (2010)	5; 3 F:2 M; 10.1–13.2 yr	AOB	NA	NA	5; 2 F:3 M; 10.1–13.2 yr	1- MOBF 2- EMG	Moderate

Abbreviations: ?, no information; ant., anterior; AOB, anterior open bite; AXB, anterior cross bite; BPXB, bilateral posterior crossbite; CI, angle’s classification; EMG, electromyography; F, females; FA, fixed appliance; HG, head gear; M, males; mo, month; yr, year; *N*, sample size; NA, not applicable; QH, quad-helix; tx, treatment; UAXB, unilateral anterior cross bite; UPXB, unilateral posterior crossbite; XP, crossbite

**Table 2:** The main results of the four chewing parameters of children with different types of malocclusions compared to children with normal occlusion

Parameter	Dentition	Sub-parameter	Cl I	Cl II	Cl III	PXB	AXB	Crowding	Long face	AOB	Deep bite	After treatment
MOBF	Primary	NA	NA	NA	NA	= [28, 36, 37]	NA	NA	NA	= [36]	NA	NA
	Mixed/permanent	NA	↓[29, 31]	↓[29, 31]	NA	↓[28, 30, 37]	NA	NA	= [59]	= [48]	NA	↓[31]
EMG	Primary	Rest	NA	NA	NA	NA	NA	NA	NA	NA	NA	NA
		Maximal clench	NA	NA	NA	NA	NA	NA	NA	NA	NA	NA
		Chewing	NA	NA	NA	NA	NA	NA	NA	NA	NA	NA
	Mixed/permanent	Rest	NA	↕[61, 64, 81]	NA	= [38, 54, 56, 78]	NA	NA	NA	NA	= [60]	↕[57, 61, 80]
		Maximal clench	NA	↕[61, 64, 81]	= [76]	↕[38, 54, 56, 62]	NA	NA	NA	↓[50, 82]	= [60]	↕[57, 61, 79, 80]
		Chewing	NA	↓[65]	= [76]	= [38, 40, 58, 63, 78]	NA	= [66]	NA	↕[33, 48, 82]	NA	= [40, 55, 58]
Jaw kinematics	Primary	Chewing time	NA	NA	NA	NA	NA	NA	NA	NA	NA	NA
	Mixed/permanent		NA	= [65]	NA	↕[42, 55, 63]	NA	NA	NA	↓[33]	NA	NA
	Primary	Jaw chewing trajectory; pattern	NA	NA	NA	↑; reverse pattern [44]	↑[68]	NA	NA	NA	NA	NA
	Mixed/permanent		NA	NA	NA	↕[38, 41, 42]; reverse pattern [42, 43, 51, 55, 72, 73]	NA	NA	NA	NA	NA	= [55, 73]
Chewing efficiency	Primary	Food trituration	NA	NA	NA	↓[46]	NA	NA	NA	↓[46]	NA	NA
	Mixed/permanent		= [34, 70]	↕[22, 34, 45, 70]	↓[34, 70]	NA	NA	NA	NA	NA	NA	↓[22]
	Primary	Mixing ability	NA	NA	NA	NA	NA	NA	NA	NA	NA	NA
	Mixed/permanent		NA	NA	NA	NA	NA	NA	NA	↕[74, 75]	NA	NA

Abbreviations: *Cl I, Cl II, and Cl III*, class I, II, and III malocclusions; PXB, posterior (unilateral and bilateral) crossbite; AXB, anterior crossbite; AOB, anterior open bite; NA, not applicable; Equal ( =), reduced (↓) or increased (↑) parameter activity in children with malocclusion than controls; ↕, conflicting results

### Maximum occlusal bite force

There were eleven studies that investigated the MOBF in children with malocclusion [28–31, 36, 37, 47–49, 59, 70]. Specifically, three studies examined children aged 3–7 years [28, 36, 37], while the remaining studies examined older children [29–31, 48, 59, 70]. MOBF was mostly compared between children with or without unilateral posterior crossbite (UPXB) [28, 30, 36, 37] or between children with or without anterior open bite (AOB) [36, 48]. Further, two studies compared MOBF between children with class I, class II, and/or class III malocclusion and children with normal occlusion [29, 70]. Three studies compared MOBF in children with class II division I malocclusion before and after functional orthodontic treatment [31, 47, 49], while one study investigated the effect of increased vertical face dimension on MOBF [59].

Children with UPXB [28, 36, 37] or with AOB [36] in the primary dentition had similar MOBF compared to children with normal occlusion. During the mixed/permanent dentition stage, the magnitude of MOBF increased in children with malocclusion [29, 30, 70], similar to a previous meta-analysis of children with normal occlusion [23]. Children with UPXB appear to have similar MOBF on their crossbite and non-crossbite sides [30] but displayed a significantly lower MOBF than crossbite-free children [28, 30, 37], while children with AOB had a similar MOBF to AOB-free controls [48]. The MOBF in children with a class II/1 malocclusion increased 1 to 2 years before orthodontic functional treatment [49] but decreased during treatment [47, 49]. After 1 year of retention, the MOBF in children with class II/1 increased and reached bite force values before functional orthodontic treatment [47]. When MOBF in children with class I or class II/1 malocclusion was compared with children with normal occlusion, it was found to be lower [29, 31]. This trend remained until after the malocclusion was orthodontically corrected with functional appliances [31]. No relationship was observed between the MOBF and vertical facial dimension [59] or between MOBF and gonial angle or masseter muscle thickness [31], but children with a class II/1 malocclusion who had the lowest MOBF showed more favorable treatment outcomes than children with a higher MOBF [49]. The overall quality of evidence regarding the effect of orthodontic treatment on MOBF in children with Cl II/1 malocclusion is “moderate” (Supplementary file 3) due to the lack of control groups in two studies [47, 49].

**Fig. 2:**
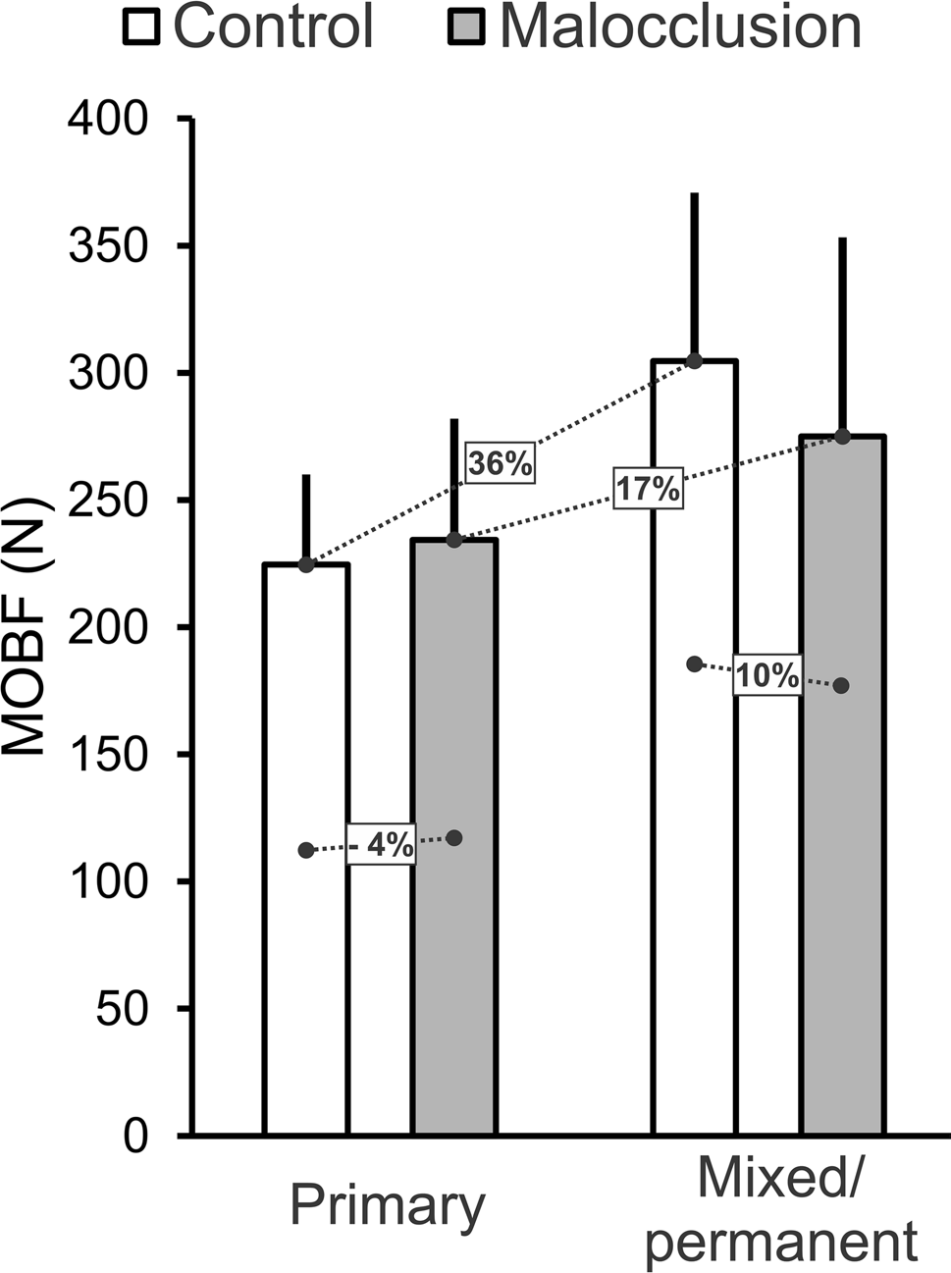
The pooled maximum occlusal bite force (SD) of children with malocclusion compared to malocclusion-free children during the primary and mixed/permanent dentition stages. The figure also shows the relative differences of MOBF between controls and children with malocclusion during the two dentition stages and between the same group during the two dentition stages

Figure 2 shows the pooled MOBF from seven studies [28, 30, 31, 36, 37, 48, 59] of children with normal occlusion compared to children with malocclusion during primary and mixed/permanent dentition stages. The relative difference of MOBF between children with normal occlusion and children with malocclusion was low (− 4%) during the primary dentition. During mixed/permanent dentition, both groups showed an increase in MOBF, but the magnitude of increase for the control group (38%) was twice that of the malocclusion group (17%), resulting in a greater relative difference between the two groups during mixed/permanent dentition (10%).

### Electromyography during chewing action

The EMG activity of the masticatory muscles was investigated in 30 studies, mostly in children in mixed/permanent dentition stage [32, 33, 35, 38–40, 48, 50, 52–58, 60–67, 76–82]. The EMG activity of masseter and anterior temporalis muscles was investigated in relation to the Dental Health Component of the Index of Orthodontic Treatment Need (IOTN-DHC) [77], or compared between children with or without AOB [33, 48, 50, 82], with or without an anterior deep bite [60], or with or without teeth crowding [66]. Furthermore, the EMG activity of masseter and/or temporalis muscles was investigated in children with class I [32, 64], class II [32, 64, 65], or class III malocclusions [32, 76] or with or without incompetent lips [35]. The masseter and temporalis EMG activity was also compared before and after functional orthodontic treatment of children with class II/1 [57, 61, 79], class II/2 [81], or class III malocclusion [80]. Studies also examined the EMG activity of the masseter and temporalis muscles of children with untreated unilateral/bilateral buccal crossbite [39, 53, 54, 56, 62, 63, 78] or before and after orthodontic crossbite treatment with quad-helix [38, 52], Rapid Maxillary Expansion (RME) [40, 58, 67] or “function generating bite” functional appliance [55], and/or after 6 months of RME treatment retention [58, 67].

The EMG activity among the studies was investigated during mandibular resting [32, 35, 38, 48, 50, 52–54, 56, 60–62, 64, 65, 77, 78, 80, 81], lateral excursion [77], protrusion [77], maximal clenching [33, 38, 48, 50, 52–54, 56–58, 60–62, 64, 66, 67, 76, 77, 79–82], or chewing behaviors [33, 38–40, 48, 55, 58, 63, 65, 66, 76–78, 82].

Similar EMG activity of masseter and temporalis muscles was observed in children with no, few, or slight-to-borderline orthodontic treatment needs [77], in children with or without teeth crowding [66], or in children with or without deep bite [60]. Lip incompetency in adolescents resulted in lower EMG resting activity of anterior temporalis compared to adolescents with competent lips [35]. For children with AOB, one study showed that they had lower EMG activity of masseter and anterior temporalis muscles compared to AOB-free children during chewing hard food [33], while another study did not [48]. Furthermore, two studies showed that children with skeletal AOB showed lower clenching and chewing EMG activities of masseter and temporalis muscles compared to AOB-free children [50, 82] and/or children with dentoalveolar AOB [82]. Both AOB-free children and children with dentoalveolar AOB were able to adapt their EMG activity during clenching or chewing activities, while children with skeletal AOB did not [82]. Orthodontic myofunctional therapy of children with skeletal AOB increased their EMG activity of masseter and anterior temporalis muscles during maximal clenching [50].

The resting EMG activity of masseter muscle showed an age-related increase in children with class I malocclusion but not with class II and III malocclusions [32]. The resting and clenching EMG activities between untreated children with class I and class II malocclusions yielded no differences in two studies [61, 64], but they were lower in children with class II malocclusion in one study [81]. Furthermore, the chewing EMG activity was lower in children with class II/1 [65] but not with class III malocclusion [76] compared to malocclusion-free children. Contradicting results were reported on the changes in EMG activity in relation to orthodontic treatment of children with malocclusions. While others found that resting [61, 79, 80] or clenching [79, 80] EMG activities of masseter and temporalis muscles in children with class I, class II/1, or class III malocclusions were stable, regardless of treatment type. Others found that functional orthodontic treatment of children with class II/1 and/or II/2 malocclusions resulted in an increase [57, 81] or decrease [61] in the masseter and temporalis muscle EMG activity during mandibular clenching, which reached control levels after treatment [57, 81].

No sex-related differences were observed in children with functional UPXB at mandibular resting, but at mandibular clenching, boys showed more EMG activity [53]. Compared to crossbite-free children, children with UPXB displayed similar masseter and anterior temporalis EMG activity during mandibular resting in four studies [38, 54, 56, 78], while one study showed lower resting EMG activity for the masseter but not the anterior temporalis muscle in children with UPXB than controls [62]. During chewing activity, limited differences in masticatory muscle EMG activity exist between children with or without bilateral or UPXB [38, 40, 58, 63, 78]. Contradicting results were observed during mandibular clenching. Three studies showed lower EMG activity in UPXB children than controls for the masseter muscle only on the crossbite side [38, 54] or for both the anterior temporalis and masseter EMG activity [62], while another study showed higher anterior temporalis EMG activity in only right-sided crossbite children compared to controls [56].

**Fig. 3:**
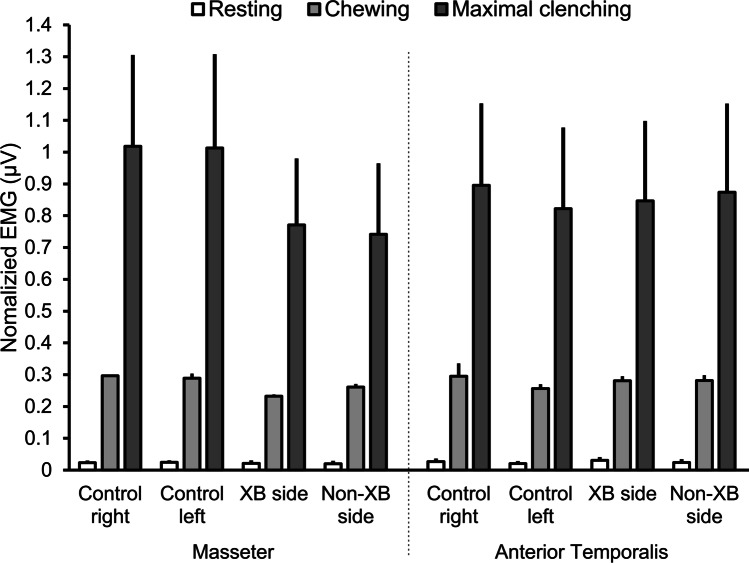
The averaged (standard error of the mean) of the normalized masseter and anterior temporalis muscle EMG activity of children with UPXB compared to crossbite-free children during mandibular resting, chewing, and maximal clenching positions. The result from each group during each behavior, muscle and side was divided (normalized) to the total average of EMG activity of all the muscles and groups combined

The bilateral EMG activation in children with UPXB compared to controls at mandibular rest was highly asymmetric in one study [52], but not in another study [56]. During clenching, children with UPXB showed symmetric EMG activity in one study [54] but two studies showed higher EMG asymmetry [52, 56], which decreases to normal control levels after orthodontic treatment [52]. During chewing, two studies reported higher masseter and/or anterior temporalis EMG asymmetry in children with UPXB compared to controls [39, 55], while three studies did not [58, 62, 63]. Moreover, the combined EMG activity of masseter and anterior temporalis muscles in children with UPXB decreased after orthodontic treatment with RME [40, 58, 67] but increased after 3–6 months of retention to resume normal levels [40, 55, 58]. Figure 3 shows the normalized EMG activity of masseter and anterior temporalis muscles in children with UPXB during the mixed/permanent dentition compared to crossbite-free children [38, 54, 56, 62, 63, 78] during mandibular resting, chewing and maximal clenching behaviors. It is evident that no difference exists on EMG activity between the two groups during mandibular resting, but perhaps a small difference is observed during chewing. During maximal clenching, however, a clear EMG difference is observed, particularly on the masseter muscle, where children with UPXB showed lower EMG activity than crossbite-free children, corroborating the observed results of MOBF (Fig. 1).

Similar to MOBF, the craniofacial parameters, such as gonial angle, showed no clear correlation to the EMG activity of masticatory muscles [60, 64, 65]. Furthermore, according to GRADE assessment, the quality of evidence regarding the effect of orthodontic treatment on EMG activity was “moderate” and “very low” for children with UPXB and Cl I, II, and III malocclusions, respectively (Supplementary file 4).

### Jaw kinematics during mastication

The jaw kinematics during chewing, such as number or duration of chewing cycles, jaw chewing pattern, or jaw lateral and vertical displacement were investigated in 16 studies [33, 38, 41–44, 51, 55, 63, 65, 67–69, 71–73]. Children with primary dentition were investigated in two studies [44, 68], while the remaining studies investigated children with mixed/permanent dentition.

The majority of the studies compared the jaw kinematics during chewing between children with untreated UPXB and crossbite-free children [41, 43, 44, 63], before and after orthodontic treatment [38, 42, 55, 67, 69, 73], or after a short-term retention [38, 51, 67]. The jaw kinematics during chewing were also compared between children with or without AOB [33], between children with or without class II/1 malocclusion [65], between children with various types of malocclusions compared to malocclusion-free children [72], or between children with or without anterior crossbite before [68, 71] and/or after orthodontic treatment [71].

No differences were observed on number of chewing cycles between children with or without class II/1 malocclusion [65]. Children with AOB while eating hard food showed shorter total chewing time and jaw closing duration with a narrower chewing cycle width than their controls [33]. Children with true anterior crossbite in the primary dentition chewed with a greater jaw sagittal opening angle than crossbite-free children [68]. Furthermore, children with UPXB in the primary dentition displayed wider jaw closing angle with higher frequency of reverse chewing cycle, particularly on the crossbite side, than crossbite-free children [44]. During the mixed/permanent dentition, the chewing cycle duration and speed in children with UPXB were similar to crossbite-free controls in two studies [55, 63], while it was slower in one study [42]. Yet their jaw closing angle remained wider than the controls, particularly on the crossbite side [55], which was restored after orthodontic intervention [55]. Furthermore, children with UPXB compared to controls [42, 43, 51, 55, 72, 73] or compared to children with anterior crossbite [43] displayed higher frequency of reverse chewing cycle, particularly on the crossbite side. However, the occurrence of reverse chewing cycle after orthodontic intervention became similar to crossbite-free controls [55, 73]. Furthermore, children with various types of malocclusions, especially who suffer from functional displacements [72] or who had anterior crossbite [71], showed a greater variability in jaw chewing pattern compared to controls. The variability of jaw chewing pattern decreased after orthodontic treatment in children with anterior crossbite and became similar to controls [71] but remained higher than the controls in children with UPXB [69]. The lateral and vertical jaw amplitude during chewing was similar in children with UPXB and crossbite-free children [41]. Another study showed that the lateral and vertical amplitude of the jaw during chewing was shorter in children with UPXB compared to crossbite-free children, which increased significantly after orthodontic treatment and remained after 1 year of retention [38]. While another study found that children with UPXB chewed with shorter vertical amplitude on the crossbite side and longer on the non-crossbite side than the crossbite-free controls, which resume normal control levels after orthodontic intervention [42]. RME treatment of children with UPXB led to an increase in maximum mouth opening [67]. Furthermore, according to GRADE assessment, the quality of evidence regarding the effect of orthodontic treatment on jaw kinematics in children with UPXB and AXB was “moderate” and “low,” respectively (Supplementary file 5).

### Chewing performance and efficiency

Eight studies investigated the chewing efficiency by measuring the food trituration and mixing ability in children with malocclusion [22, 27, 34, 45, 46, 70, 74, 75]. While one study included children in the primary dentition [46], the majority of the studies included children in the mixed/permanent dentition [22, 27, 34, 45, 70, 74, 75]. Five studies used artificial test food models as a form of silicon-based tablets (i.e., CutterSil, Optocal or Optosil tablets) [22, 27, 34, 45, 46], one study used peanuts and carrots [70], and two studies investigated food mixing ability using colorimetric capsules, which were freely masticated and their content were analyzed with a spectrophotometer [74, 75].

Various types of malocclusions were also investigated. The masticatory efficiency of children with normal occlusion was compared to children with class I, II, or III malocclusions [22, 27, 34, 45, 70], to children with AOB [46, 74, 75], or with anterior/posterior crossbite [46].

During primary dentition, the habitual chewing of children with AOB compared to children with UPXB showed no differences in the size or number of test food particles [46]. However, children with normal occlusion, compared to the two malocclusion groups, chewed the test food into greater number of particles with a smaller size [46]. Using similar colorimetric method, one study showed reduced mixing ability in children with AOB in the mixed/permanent dentition compared to AOB-free children [74], while another study did not [75]. The median particle size of both natural and artificial test food was markedly reduced with the increase in age, where children with class I and II malocclusions, except class III malocclusion, showed similar median particle size compared to children with normal occlusion [27, 34, 70].

In counter to these studies, it was shown that adolescent girls (11 to 15 years) with class II malocclusion, compared to adolescent girls with normal occlusion, had reduced chewing efficiency [45]. Similar result trend was observed after a 2-year follow-up of adolescent girls (11 to 15 years) with or without malocclusion [22]. Apart from the age-related “maturity” of the chewing efficiency in girls with or without malocclusion, it was shown that girls who have class II malocclusion, which was corrected orthodontically, showed similar chewing efficacy to girls with untreated class II malocclusion. However, both the groups of adolescent girls showed reduced chewing efficiency compared to girls with normal occlusion [22]. In addition, GRADE assessment indicated that the quality of evidence concerning the effect of orthodontic treatment on chewing efficiency for children with Cl II malocclusion was “low” (Supplementary file 6).

## Discussion

Recently, it has been suggested that objective indicators of masticatory function in children aged 10 to 14 years show a transition to an “adult type” of chewing behavior [23]. Therefore, the current systematic review examined the influence of various malocclusions on the objective indicators of chewing and jaw function in growing children. The current systematic review also examined the ability of orthodontic treatment to influence/restore abnormal jaw function. The results of the current study showed distinct differences in objective indicators of chewing and jaw function in children with and without malocclusion. Specifically, the results showed that the children group with malocclusion had similar MOBF but lower masticatory efficiency [46] than the group without malocclusion in the deciduous dentition phase [28, 36, 37]. In addition, the children group with UPXB in the primary dentition showed a wider jaw opening angle and a higher frequency of reverse chewing cycle [44] than the group without crossbite. During the mixed or permanent dentition, children with malocclusions showed lower MOBF [28, 30, 37], lower EMG activity [38, 40, 54, 56, 58, 62, 63, 78], and lower chewing efficiency [22, 34, 45, 70] compared to malocclusion-free group. Furthermore, children with UPXB in the mixed/permanent dentition also exhibited a higher frequency of reverse chewing cycle [42, 43, 51, 55, 72, 73]. The results also indicate that orthodontic treatment can generally restore normal jaw function in children with malocclusion. Specifically, orthodontic treatment reduces the frequency of the reverse chewing cycle [55, 73] and normalizes the EMG activity of the jaw muscles during chewing [57, 81]. However, the quality of evidence on the influence of orthodontic treatment on the parameters of jaw function in children with malocclusions is either “moderate” or “low.” Thus, further studies are needed to determine whether orthodontic treatment can improve jaw function and masticatory function in general.

Normal jaw function depends on a harmonious relationship between the different components of the masticatory system. This harmonious relationship may be perturbed/disrupted in children with malocclusion, which may affect the normal development and function of the jaw [83]. Besides, it is well documented that malocclusion can affect orofacial esthetic perception [84, 85], oral functional capability [86], and psychological well-being [87] of affected individuals, thus influencing their oral health related quality of life [20].

Bite force is an important indicator of the functional state of the masticatory system [88]. The results of the study show that the bite force (MOBF) is influenced by malocclusion. However, the effect of malocclusion on bite force is related to the age and dental status of children (Fig. 2). Accordingly, children with and without malocclusion in the primary dentition showed similar MOBF. However, in the mixed/permanent dentition phase, children with malocclusion showed lower bite force than children without malocclusions (control group). It has been previously shown that younger children show signs of “immature” jaw function, reflected in lower and more variable bite forces compared to older children (for review, see [23]). This observation could be explained by the immaturity of orofacial structures and lower muscle force production in children with primary dentition. It is suggested that the variability of jaw function that prevails in children with primary dentition could mask any perturbances that might occur due to malocclusion. However, as children grow and orofacial structures mature, the variability of jaw function decreases, and a clear demarcation of jaw function between children with and without malocclusion can be observed.

Orofacial deformities and malocclusions are thought to have varying degrees of impact on esthetics and masticatory function [20, 86, 88, 89]. It has also been observed that certain types of malocclusions such as UPXB could have a greater impact on normal jaw function than other types of malocclusions. Posterior crossbite is a common malocclusion affecting approximately 8% to 22% of orthodontic patients in the primary and early mixed dentition, with approximately 50% of cases being unilateral posterior crossbite (i.e., UPXB) [90, 91]. Apart from faulty dental occlusion, children with UPXB often suffer from a morphological asymmetry of the mandible [52, 92] or bilateral differences in masticatory muscle thickness [93]. These asymmetries often result in mandibular displacement during jaw function, thus affecting jaw muscle alignment and jaw muscle activity [38, 40, 54, 56, 58, 63, 78]. Although these changes do not severely affect jaw function in younger children, the jaw muscles functionally adapt to the abnormal mandibular position with age leading to changes in their muscle thickness, especially on the side of the crossbite [94]. These suggestions are also supported by findings from early animal studies that show morphological differences in areas of jaw muscle insertions, (local) bone remodeling, and changes in muscle fiber type and composition in rats fed with a hard or soft diet [95]. Similarly, EMG activity of the orofacial muscles was lower in children with incompetent lips compared to children with competent lips or in children with AOB compared to AOB-free children [35, 50, 82]. However, there are no appreciable differences in the EMG activity of the jaw muscles between children with or without crowding or with or without deep bite [60, 66]. Therefore, it is suggested that the type and severity of malocclusion may have a specific influence on normal jaw function (see Table 2).

As mentioned earlier, studies have reported that posterior malocclusions may have a greater impact on normal jaw function than anterior malocclusions. Since the posterior teeth are responsible for crushing, breaking down, and grinding food, a posterior malocclusion such as UPXB is more likely to affect oral functions, especially chewing [43, 96]. Although the anterior teeth are important in the initial stages of biting, food chewing is highly dependent on adequate occlusion of the posterior teeth [23]. Unlike anterior malocclusions, posterior malocclusions such as UPXB destabilize normal tooth occlusion and jaw muscle orientation/alignment, which can severely affect normal chewing function by reducing the efficiency of food grinding. It has already been shown that impaired chewing function could also affect the processes of swallowing and digestion. Although orthodontic correction of all types of dental malocclusions has significant esthetic and psychological benefits [20], the treatment of posterior malocclusions in particular could be of great importance in restoring normal chewing function [19].

According to longitudinal studies, a number of malocclusions (depending on their severity) may change during the period between the primary and permanent dentition [97, 98]. In other words, a malocclusion in the primary dentition may normalize spontaneously (without intervention) during growth, while other malocclusions may develop simultaneously [97]. But in most “self-uncorrected” cases and depending on the severity of the malocclusions, an orthodontic intervention may be needed. In the current study, the effects of orthodontic intervention on jaw function were evaluated in 20 studies (Table 1). Studies have shown that children who received functional appliance show “more favorable” treatment outcomes [31, 47, 49]. Orthodontic treatment of functional UPXB has been shown to correct associated morphological asymmetries and restore normal jaw function in affected children [52, 92, 93]. However, the results of the GRADE assessments showed an overall moderate to low quality of evidence for the effects of orthodontic treatment on the selected parameters of jaw motor function and mastication. Orthodontic interventions in UPXB resolved the wider jaw closing angle and significantly decreased the reverse chewing cycles during mastication [51, 55, 73]. It has also been suggested that certain aspects of masticatory kinematics respond better to orthodontic treatment than others [42]. Nevertheless, studies suggest that while self-perceived chewing ability increases after orthodontic interventions, objective chewing performance does not [22].

In the present study, the quality of evidence on the influence of orthodontic treatment on MOBF, jaw kinematics, EMG activity of jaw muscles, and chewing efficiency/performance in children with malocclusion was assessed using GRADE. The quality of evidence for all parameters was rated as “moderate” to “low.” The lower GRADE scores for quality of evidence were mainly due to the lack of well-controlled studies and studies that were unable to identify confounding factors. The lack of measures to deal with confounding factors and the heterogeneity of study samples, subgroups, and exposures also influenced the quality of evidence score. Indirectness due to substantial differences between the population, intervention, or outcomes measured in the relevant studies also influenced the outcome of quality of evidence. Therefore, orthodontic interventions have a greater impact on patient quality of life in addition to restoring esthetics and anatomy, but more robust longitudinal studies are needed to determine whether they improve masticatory function.

The causes of dental malocclusion are, of course, multifactorial, but one of the mechanisms responsible for impaired chewing function in patients requiring orthodontic treatment may be due to impaired sensorimotor regulation associated with chewing. Therefore, it may be important for clinicians to perform a functional assessment in children in whom orthodontic treatment is indicated and identify specific impairments in chewing function. Assessment of oral function, such as bite force or chewing efficiency/performance, may also help to evaluate treatment success after orthodontic interventions. Comprehensive orthodontic treatment, including evaluations and restoration of function, can mitigate the effects of malocclusion and restore normal chewing function.

## Limitations

It is recommended that the results of this review be interpreted with caution due to the quality assessment outcomes and the methodological heterogeneity of the studies. Specifically, twenty-six studies included in the current systematic review were classified as low quality, which may call into question the accuracy/validity of the findings. In addition, with the exception of two studies [27, 34], the studies included in this review examined different malocclusions classified according to their class or three-dimensional characteristics without controlling for the severity of malocclusion within each classification. This may lead to discrepancies in the interpretation of the results in the included studies. It is known that other factors, such as those related to individual participant variation or differences in the experimental protocol, may also affect normal jaw function. It has been previously shown that normal jaw function can be influenced by children’s age, body size, tooth contact area, and bite forces [23, 27]. Other experimental factors include variations in dietary characteristics [99] and a lack of normalization protocol for jaw function measurement devices [100]. Thus, failure to control the above factors could lead to variations in jaw function outcomes in children with malocclusion. However, the results of the current study were carefully pooled based on the age and dentition stage of the patients (primary versus mixed/permanent) as illustrated in Fig. 2. Furthermore, the results of studies with similar methodologies and malocclusion groups were also normalized to allow for accurate and meaningful comparisons (see Fig. 3). Therefore, the current systematic review provides an overview of the current literature.

## Conclusions

According to the limitations of the studies included, it is not possible to confirm or deny the existence of an association between dental/skeletal malocclusion traits and MOBF, EMG, jaw kinematics, and masticatory performance in healthy children. The results also highlight the role of orthodontic treatment in the restoration of normal jaw function in affected children. However, the absence of well-controlled and well-designed studies, nevertheless, makes it impossible to conclude whether comprehensive orthodontic treatment, which includes evaluation and restoration of function, may/may not mitigate the effects of malocclusion, and restore normal jaw motor and chewing function.

## Supplementary Information

Below is the link to the electronic supplementary material.

Supplementary file1 (PDF 46 KB)

Supplementary file2 (DOCX 30 KB)

Supplementary file3 (DOC 55 KB)

Supplementary file4 (DOC 56 KB)

Supplementary file5 (DOC 54 KB)

Supplementary file6 (DOC 54 KB)

Supplementary file7 (DOCX 32 KB)

## References

[1] Lund JP (1991). Mastication and its control by the brain stem. Crit Rev Oral Biol Med.

[2] Proff P (2010). Malocclusion, mastication and the gastrointestinal system. J Orofac Orthop.

[3] Grigoriadis A, Kumar A, Åberg MK, Trulsson M (2019). Effect of sudden deprivation of sensory inputs from periodontium on mastication. Front Neurosci.

[4] Sessle BJ, Avivi-Arber L, Murray GM, McLoon L, Andrade F (2012). Motor control of masticatory muscles. Craniofacial Muscles.

[5] Trulsson M (2006). Sensory-motor function of human periodontal mechanoreceptors. J Oral Rehabil.

[6] van der Bilt A, Engelen L, Pereira LJ, van der Glas HW, Abbink JH (2006). Oral physiology and mastication. Physiol Behav.

[7] Chen J (2009). Food oral processing—a review. Food Hydrocoll.

[8] Dodds M, Roland S, Edgar M, Thornhill M (2015). Saliva A review of its role in maintaining oral health and preventing dental disease. BDJ Team.

[9] Sheiham A, Steele J (2001). Does the condition of the mouth and teeth affect the ability to eat certain foods, nutrient and dietary intake and nutritional status amongst older people?. Public Health Nutr.

[10] Watson S, McGowan L, McCrum LA, Cardwell CR, McGuinness B, Moore C, Woodside JV, McKenna G (2019). The impact of dental status on perceived ability to eat certain foods and nutrient intakes in older adults: cross-sectional analysis of the UK National Diet and Nutrition Survey 2008–2014. Int J Behav Nutr Phys Act.

[11] Brennan DS, Spencer AJ, Roberts-Thomson KF (2008). Tooth loss, chewing ability and quality of life. Qual Life Res.

[12] Kumar A, Kothari M, Grigoriadis A, Trulsson M, Svensson P (2018). Bite or brain: Implication of sensorimotor regulation and neuroplasticity in oral rehabilitation procedures. J Oral Rehabil.

[13] Abrahamsson C (2013). Masticatory function and temporomandibular disorders in patients with dentofacial deformities. Swed Dent J Suppl.

[14] Kiliaridis S, Karlsson S, Kjellberg H (1991). Characteristics of masticatory mandibular movements and velocity in growing individuals and young adults. J Dent Res.

[15] Saitoh I, Hayasaki H, Nakata S, Iwase Y, Nakata M (2004). Characteristics of the gum chewing occlusal phase in children with primary dentition. J Oral Rehabil.

[16] Almotairy N, Kumar A, Noirrit-Esclassan E, Grigoriadis A (2020). Developmental and age-related changes in sensorimotor regulation of biting maneuvers in humans. Physiol Behav.

[17] Almotairy N, Kumar A, Grigoriadis A (2020). Effect of food hardness on chewing behavior in children. Clin Oral Investig.

[18] Almotairy N, Kumar A, Welander N, Grigoriadis A (2020). Age-related changes in oral motor-control strategies during unpredictable load demands in humans. Eur J Oral Sci.

[19] Magalhães IB, Pereira LJ, Marques LS, Gameiro GH (2010). The influence of malocclusion on masticatory performance a systematic review. Angle Orthod.

[20] Dimberg L, Arnrup K, Bondemark L (2015). The impact of malocclusion on the quality of life among children and adolescents: a systematic review of quantitative studies. Eur J Orthod.

[21] Pepicelli A, Woods M, Briggs C (2005). The mandibular muscles and their importance in orthodontics: A contemporary review. Am J Orthod Dentofac Orthop.

[22] Henrikson T, Ekberg E, Nilner M (2009). Can orthodontic treatment improve mastication? A controlled, prospective and longitudinal study. Swed Dent J.

[23] Almotairy N, Kumar A, Trulsson M, Grigoriadis A (2018). Development of the jaw sensorimotor control and chewing - a systematic review. Physiol Behav.

[24] Page MJ, McKenzie JE, Bossuyt PM, Boutron I, Hoffmann TC, Mulrow CD, Shamseer L, Tetzlaff JM, Akl EA, Brennan SE, Chou R, Glanville J, Grimshaw JM, Hróbjartsson A, Lalu MM, Li T, Loder EW, Mayo-Wilson E, McDonald S, McGuinness LA, Stewart LA, Thomas J, Tricco AC, Welch VA, Whiting P, Moher D (2021). The PRISMA 2020 statement: an updated guideline for reporting systematic reviews. BMJ.

[25] Moola S, Munn Z, Tufanaru C, Aromataris E, Sears K, Sfetcu R, Currie M, Qureshi R, Mattis P, Lisy K, Mu P-F (2017) Checklist for Analytical cross sectional studies. In: Aromataris E, Munn Z (eds) Joanna Briggs Institute Reviewer’s Manual. The Joanna Briggs Institute, Adelaide, p 6

[26] [Software] GradeGGradeGDT (2021) McMaster University and Evidence Prime. gradepro.org

[27] Toro A, Buschang PH, Throckmorton G, Roldán S (2006). Masticatory performance in children and adolescents with class I and II malocclusions. Eur J Orthod.

[28] Castelo PM, Gavião MBD, Pereira LJ, Bonjardim LR (2010). Maximal bite force, facial morphology and sucking habits in young children with functional posterior crossbite. J Appl Oral Sci.

[29] Roldan SI, Restrepo LG, Isaza JF, Velez LG, Buschang PH (2016). Are maximum bite forces of subjects 7 to 17 years of age related to malocclusion?. Angle Orthod.

[30] Sonnesen L, Bakke M, Solow B (2001). Bite force in pre-orthodontic children with unilateral crossbite. Eur J Orthod.

[31] Antonarakis GS, Kiliaridis S (2015). Predictive value of masseter muscle thickness and bite force on Class II functional appliance treatment: a prospective controlled study. Eur J Orthod.

[32] Sabashi K, Saitoh I, Hayasaki H, Iwase Y, Kondo S, Inada E, Takemoto Y, Yamada C, Yamasaki Y (2009). A cross-sectional study of developing resting masseter activity in different angle classifications in adolescence. Cranio.

[33] Piancino MG, Isola G, Merlo A, Dalessandri D, Debernardi C, Bracco P (2012). Chewing pattern and muscular activation in open bite patients. J Electromyogr Kinesiol.

[34] Barrera LM, Buschang PH, Throckmorton GS, Roldan SI (2011). Mixed longitudinal evaluation of masticatory performance in children 6 to 17 years of age. Am J Orthod Dentofac Orthop.

[35] Lipari MA, Pimentel G, Gamboa NA, Bayas I, Guerrero N, Miralles R (2020). Electromyographic comparison of lips and jaw muscles between children with competent and incompetent lips: a cross sectional study. J Clin Pediatr Dent.

[36] Rentes A, Gaviao M, Amaral J (2002). Bite force determination in children with primary dentition. J Oral Rehabil.

[37] Castelo PM, Gavião MBD, Pereira LJ, Bonjardim LR (2007). Masticatory muscle thickness, bite force, and occlusal contacts in young children with unilateral posterior crossbite. Eur J Orthod.

[38] Martín C, Palma JC, Alamán JM, Lopez-Quiñones JM, Alarcón JA (2012). Longitudinal evaluation of sEMG of masticatory muscles and kinematics of mandible changes in children treated for unilateral cross-bite. J Electromyogr Kinesiol.

[39] Ferrario VF, Sforza C, Serrao G (1999). The influence of crossbite on the coordinated electromyographic activity of human masticatory muscles during mastication. J Oral Rehabil.

[40] Spolaor F, Mason M, De Stefani A, Bruno G, Surace O, Guiotto A, Gracco A, Sawacha Z (2020). Effects of rapid palatal expansion on chewing biomechanics in children with malocclusion: a surface electromyography study. Sensors (Basel).

[41] Salioni MA, Pellizoni SE, Guimaraes AS, Juliano Y, Alonso LG (2005). Functional unilateral posterior crossbite effects on mastication movements using axiography. Angle Orthod.

[42] Throckmorton GS, Buschang PH, Hayasaki H, Pinto AS (2001). Changes in the masticatory cycle following treatment of posterior unilateral crossbite in children. Am J Orthod Dentofac Orthop.

[43] Piancino MG, Comino E, Talpone F, Vallelonga T, Frongia G, Bracco P (2012). Reverse-sequencing chewing patterns evaluation in anterior versus posterior unilateral crossbite patients. Eur J Orthod.

[44] Sever E, Marion L, Ovsenik M (2011). Relationship between masticatory cycle morphology and unilateral crossbite in the primary dentition. Eur J Orthod.

[45] Henrikson T, Ekberg EC, Nilner M (1998). Masticatory efficiency and ability in relation to occlusion and mandibular dysfunction in girls. Int J Prosthodont.

[46] Beatriz Duarte Gavião M, Graciele Raymundo V, LourençoCorrer Sobrinho D (2001). Masticatory efficiency in children with primary dentition. Pediatr Dent.

[47] Antonarakis GS, Kjellberg H, Kiliaridis S (2013). Bite force and its association with stability following Class II/1 functional appliance treatment. Eur J Orthod.

[48] Yousefzadeh F, Shcherbatyy V, King GJ, Huang GJ, Liu ZJ (2010). Cephalometric and electromyographic study of patients of East African ethnicity with and without anterior open bite. Am J Orthod Dentofac Orthop.

[49] Antonarakis GS, Kjellberg H, Kiliaridis S (2012). Predictive value of molar bite force on Class II functional appliance treatment outcomes. Eur J Orthod.

[50] Hong H, Zeng Y, Chen X, Peng C, Deng J, Zhang X, Deng L, Xie Y, Wu L (2021). Electromyographic features and efficacy of orofacial myofunctional treatment for skeletal anterior open bite in adolescents: an exploratory study. BMC Oral Health.

[51] Ben-Bassat Y, Yaffe A, Brin I, Freeman J, Ehrlich Y (1993). Functional and morphological-occlusal aspects in children treated for unilateral posterior cross-bite. Eur J Orthod.

[52] Kecik D, Kocadereli I, Saatci I (2007). Evaluation of the treatment changes of functional posterior crossbite in the mixed dentition. Am J Orthod Dentofac Orthop.

[53] Lenguas L, Alarcón J-A, Venancio F, Kassem M, Martín C (2012). Surface electromyographic evaluation of jaw muscles in children with unilateral crossbite and lateral shift in the early mixed dentition. Sexual dimorphism. Med Oral Patol Oral Cir Bucal.

[54] Alarcón JA, Martín C, Palma JC, Menéndez-Núñez M (2009). Activity of jaw muscles in unilateral cross-bite without mandibular shift. Arch Oral Biol.

[55] Piancino MG, Falla D, Merlo A, Vallelonga T, de Biase C, Dalessandri D, Debernardi C (2016). Effects of therapy on masseter activity and chewing kinematics in patients with unilateral posterior crossbite. Arch Oral Biol.

[56] Andrade AS, Gavião MBD, Derossi M, Gameiro GH (2009). Electromyographic activity and thickness of masticatory muscles in children with unilateral posterior crossbite. Clin Anat.

[57] Satygo EA, Silin AV, Ramirez-Yañez GO (2014). Electromyographic muscular activity improvement in Class II patients treated with the pre-orthodontic trainer. J Clin Pediatr Dent.

[58] Michelotti A, Rongo R, Valentino R, D’Antò V, Bucci R, Danzi G, Cioffi I (2019). Evaluation of masticatory muscle activity in patients with unilateral posterior crossbite before and after rapid maxillary expansion. Eur J Orthod.

[59] Proffit WR, Fields HW (1983). Occlusal Forces in Normal- and Long-face Children. J Dent Res.

[60] Takada K, Lowe AA (1985). Multiple regression analysis of craniofacial and jaw muscle variables in control and deep-bite subjects. J Osaka Univ Dent Sch.

[61] Ingervall B, Thüer U (1991). Temporal muscle activity during the first year of Class II, division 1 malocclusion treatment with an activator. Am J Orthod Dentofacial Orthop.

[62] Ciavarella D, Monsurrò A, Padricelli G, Battista G, Laino L, Perillo L (2012). Unilateral posterior crossbite in adolescents: surface electromyographic evaluation. Eur J Paediatr Dent.

[63] da Andrade A, S, Gaviao MBD, Gameiro GH, De Rossi M,  (2010). Characteristics of masticatory muscles in children with unilateral posterior crossbite. Braz Oral Res.

[64] Lowe AA, Takada K (1984). Associations between anterior temporal, masseter, and orbicularis oris muscle activity and craniofacial morphology in children. Am J Orthod.

[65] Ahlgren JG, Ingervall BF, Thilander BL (1973). Muscle activity in normal and postnormal occlusion. Am J Orthod.

[66] Hinotume S, Morinushi T, Ogura T (1994). Masticatory function in normal and crowded occlusion using Hellman’s dental stages. J Clin Pediatr Dent.

[67] Galbiati G, Maspero C, Giannini L, Tagliatesta C, Farronato G (2016). Functional evaluation in young patients undergoing orthopedical interceptive treatment. Minerva Stomatol.

[68] Nagata M, Yamasaki Y, Hayasaki H, Nakata M (2002). Incisal and condylar paths during habitual mouth opening movement of children with anterior reverse bite in the primary dentition. J Oral Rehabil.

[69] Keeling SD, Gibbs CH, Lupkiewicz SM, King GJ, Jacobson AP (1991). Analysis of repeated-measure multicycle unilateral mastication in children. Am J Orthod Dentofac Orthop.

[70] Shiere FR, Manly RS (1952). The effect of the changing dentition on masticatory function. J Dent Res.

[71] Yashiro K, Miyawaki S, Takada K (2004). Stabilization of jaw-closing movements during chewing after correction of incisor crossbite. J Oral Rehabil.

[72] Ahlgren J (1967). Pattern of chewing and malocclusion of teeth a clinical study. Acta Odontol Scand.

[73] Piancino MG, Cordero-Ricardo M, Cannavale R, Vallelonga T, Garagiola U, Merlo A (2017). Improvement of masticatory kinematic parameters after correction of unilateral posterior crossbite: Reasons for functional retention. Angle Orthod.

[74] Corrêa EC, Maeda FA, de Miranda ALR, Carvalho PEG, Da Silva LH, Torres FC (2018). Masticatory evaluation of anterior open bite malocclusion using the colorimetric capsule method. Gen Dent.

[75] Costa ES, Cazal MS, Mestriner Junior W, Pithon MM, Guimarães AS (2019). Masticatory performance between individuals with good overbite and patients with anterior open bite. J World Fed Orthod.

[76] Go Y (1981). An electromyographic study on masticatory muscles - comparison and examination of crossbite patients preoperatively, postoperatively and in post retention. J Nihon Univ Sch Dent.

[77] Hallak Regalo SC, de Lucas B, L, Diaz-Serrano KV, Ribeiro Frota NP, Regalo IH, Pereira Nassar MS, Righetti MA, Oliveira LF, Napolitano Goncalves LM, Siessere S, Palinkas M, Regalo SCH, de Lima Lucas B, Diaz-Serrano KV, Frota NPR, Regalo IH, Nassar MSP, Righetti MA, Oliveira LF, Goncalves LMN, Siessere S, Palinkas M,  (2018). Analysis of the stomatognathic system of children according orthodontic treatment needs. J Orofac Orthop.

[78] Alarcón JA, Martín C, Palma JC (2000). Effect of unilateral posterior crossbite on the electromyographic activity of human masticatory muscles. Am J Orthod Dentofac Orthop.

[79] Di Palma E, Tepedino M, Chimenti C, Tartaglia GM, Sforza C (2017). Effects of the functional orthopaedic therapy on masticatory muscles activity. J Clin Exp Dent.

[80] Nuño-Licona A, Cavazos E, Angeles-Medina F (1993). Electromyographic changes resulting from orthodontic correction of class III malocclusion. Int J Paediatr Dent.

[81] Petrović D, Vujkov S, Petronijević B, Šarčev I, Stojanac I (2014). Examination of the bioelectrical activity of the masticatory muscles during Angle’s Class II division 2 therapy with an activator. Vojnosanit Pregl.

[82] Ciccone De Faria TDS, Hallak Regalo SC, Thomazinho A, Vitti M, De Felício CM (2010). Masticatory muscle activity in children with a skeletal or dentoalveolar open bite. Eur J Orthod.

[83] English JD, Buschang PH, Throckmorton GS, J.D. E, P.H. B, G.S. T,  (2002). Does malocclusion affect masticatory performance?. Angle Orthod.

[84] Van der Geld P, Oosterveld P, Van Heck G, Kuijpers-Jagtman AM (2007). Smile Attractiveness. Angle Orthod.

[85] Janson G, Branco NC, Fernandes TMF, Sathler R, Garib D, Lauris JRP (2011). Influence of orthodontic treatment, midline position, buccal corridor and smile arc on smile attractiveness. Angle Orthod.

[86] Magalhaes IB, Pereira LJ, Marques LS, Gameiro GH (2010). The influence of malocclusion on masticatory performance a systematic review. Angle Orthod.

[87] de Paula JDF, Santos NCM, da Silva ÉT, Nunes MF, Leles CR (2009). Psychosocial impact of dental esthetics on quality of life in adolescents. Angle Orthod.

[88] Koc D, Dogan A, Bek B (2010). Bite force and influential factors on bite force measurements: a literature review. Eur J Dent.

[89] Bourdiol P, Soulier-Peigue D, Lachaze P, Nicolas E, Woda A, Hennequin M (2017). Only severe malocclusion correlates with mastication deficiency. Arch Oral Biol.

[90] Caroccia F, Moscagiuri F, Falconio L, Festa F, D’Attilio M (2020). Early Orthodontic Treatments of Unilateral Posterior Crossbite: A Systematic Review. J Clin Med.

[91] Kennedy DB, Osepchook M (2005). Unilateral posterior crossbite with mandibular shift: a review. J Can Dent Assoc.

[92] Pinto AS, Buschang PH, Throckmorton GS, Chen P (2001). Morphological and positional asymmetries of young children with functional unilateral posterior crossbite. Am J Orthod Dentofac Orthop.

[93] Kiliaridis S, Mahboubi PH, Raadsheer MC, Katsaros C (2007). Ultrasonographic Thickness of the Masseter Muscle in Growing Individuals with Unilateral Crossbite. Angle Orthod.

[94] Grünheid T, Langenbach GEJ, Korfage JAM, Zentner A, van Eijden TMGJ (2009). The adaptive response of jaw muscles to varying functional demands. Eur J Orthod.

[95] Kiliaridis S, Engström C, Thilander B (1988). Histochemical analysis of masticatory muscle in the growing rat after prolonged alteration in the consistency of the diet. Arch Oral Biol.

[96] Soares Maria Eliza Consolação, B, Letícia Ramos-Jorge M, Mota de Alencar B, Silva Marques L, José Pereira L, Ramos-Jorge J,  (2016). Factors associated with masticatory performance among preschool children. Clin Oral Investig.

[97] Dimberg L, Lennartsson B, Arnrup K, Bondemark L (2015). Prevalence and change of malocclusions from primary to early permanent dentition: a longitudinal study. Angle Orthod.

[98] Leighton BC, Feasby WH (1988). Factors influencing the development of molar occlusion: a longitudinal study. Br J Orthod.

[99] Takada K, Miyawaki S, Tatsuta M (1994). The effects of food consistency on jaw movement and posterior temporalis and inferior orbicularis oris muscle activities during chewing in children. Arch Oral Biol.

[100] Ferrario VF, Sforza C, Tartaglia GM (2009). Commentary to suvinen and kemppainen (JOR 2007;34:631–44): Commentary. J Oral Rehabil.

