# Supplementary material for: Effect of malocclusion on jaw motor function and chewing in children: a systematic review
Source: Clin Oral Investig. 2022 Jan 5;26(3):2335–51. doi: 10.1007/s00784-021-04356-y (PMC8898242; doi:10.1007/s00784-021-04356-y)
Supplement: Supplementary file 1 — Supplementary file1 (PDF 46 KB) [file 784_2021_4356_MOESM1_ESM.pdf]

1. Medline (Ovid)

1. exp Mastication/
2. Bite Force/
3. exp Masticatory Muscles/
4. or/1-3

5. Electromyography/
6. exp Biomechanical Phenomena/
7. Movement/
8. (develop* or efficienc* or behav* or rate* or function* or perform* or evaluat* or activit* or physiolog* or electromyogra* or movement* or freq* or speed* or rhythm* or kinemat* or pattern* or system* or videograph* or optic*).ti,ab,kf.
9. or/5-8

10. 4 and 9

11. ((chew* or masticat*) adj3 (develop* or efficienc* or behav* or rate* or function* or perform* or evaluat* or activit* or physiolog* or electromyogra* or movement* or freq* or speed* or rhythm* or kinemat* or pattern* or system* or videograph* or optic*)).ti,ab,kf.
12. 10 or 11

13. limit 12 to "all child (0 to 18 years)"

14. (infant* or infancy or baby* or babies or child* or schoolchild* or school age* or preschool* or kid or kids or toddler* or adoles* or teen* or boy* or girl*).ti,ab,kf.

15. 12 and 14

16. 13 or 15

17. remove duplicates from 16

18. limit 17 to english language

2. Embase (embase.com)

('mastication'/de OR 'masticatory muscle'/exp

AND

'electromyography'/de OR 'kinematics'/de OR 'biomechanics'/de OR 'movement (physiology)'/de OR develop*:ti,ab OR efficienc*:ti,ab OR behav*:ti,ab OR rate*:ti,ab OR function*:ti,ab OR perform*:ti,ab OR evaluat*:ti,ab OR activit*:ti,ab OR physiolog*:ti,ab OR electromyogra*:ti,ab OR movement*:ti,ab OR freq*:ti,ab OR speed*:ti,ab OR rhythm*:ti,ab OR kinemat*:ti,ab OR pattern*:ti,ab OR system*:ti,ab OR videograph*:ti,ab OR optic*:ti,ab

OR

((chew* OR masticat*) NEAR/3 (develop* OR efficienc* OR behav* OR rate* OR function* OR perform* OR evaluat* OR activit* OR physiolog* OR electromyogra* OR movement* OR freq* OR speed* OR rhythm* OR kinemat* OR pattern* OR system* OR videograph* OR optic*)):ti,ab

AND ([adolescent]/lim OR [child]/lim)

OR 'mastication'/de OR 'masticatory muscle'/exp

AND

'electromyography'/de OR 'kinematics'/de OR 'biomechanics'/de OR 'movement (physiology)'/de OR develop*:ti,ab OR efficienc*:ti,ab OR behav*:ti,ab OR rate*:ti,ab OR function*:ti,ab OR perform*:ti,ab OR evaluat*:ti,ab OR activit*:ti,ab OR physiolog*:ti,ab OR electromyogra*:ti,ab OR movement*:ti,ab OR freq*:ti,ab OR speed*:ti,ab OR rhythm*:ti,ab OR kinemat*:ti,ab OR pattern*:ti,ab OR system*:ti,ab OR videograph*:ti,ab OR optic*:ti,ab

OR

((chew* OR masticat*) NEAR/3 (develop* OR efficienc* OR behav* OR rate* OR function* OR perform* OR evaluat* OR activit* OR physiolog* OR electromyogra* OR movement* OR freq* OR speed* OR rhythm* OR kinemat* OR pattern* OR system* OR videograph* OR optic*)):ti,ab

AND

infant*:ti,ab OR infancy:ti,ab OR baby*:ti,ab OR babies:ti,ab OR child*:ti,ab OR schoolchild*:ti,ab OR 'school age*':ti,ab OR preschool*:ti,ab OR kid:ti,ab OR kids:ti,ab OR toddler*:ti,ab OR adoles*:ti,ab OR teen*:ti,ab OR boy*:ti,ab OR girl*:ti,ab)

AND [english]/lim

3. Web of Science Core Collection

TS=((chew* OR masticat*) NEAR/3 (develop* OR efficienc* OR behav* OR rate* OR function* OR perform* OR evaluat* OR activit* OR physiolog* OR electromyogra* OR movement* OR freq* OR speed* OR rhythm* OR kinemat* OR pattern* OR system* OR videograph* OR optic*))

AND

TS=(infant* OR infancy OR baby* OR babies OR child* OR schoolchild* OR "school age*" OR preschool* OR kid OR kids OR toddler* OR adoles* OR teen* OR boy* OR girl*)

Refined by: LANGUAGES: (ENGLISH)

Indexes=SCI-EXPANDED, SSCI, A&HCI, CPCI-S, CPCI-SSH, ESCI Timespan=All years
