# Supplementary material for: Effect of malocclusion on jaw motor function and chewing in children: a systematic review
Source: Clin Oral Investig. 2022 Jan 5;26(3):2335–51. doi: 10.1007/s00784-021-04356-y (PMC8898242; doi:10.1007/s00784-021-04356-y)
Supplement: Supplementary file 2 — Supplementary file2 (DOCX 30 KB) [file 784_2021_4356_MOESM2_ESM.docx]

**Supplemental file 2.** Detailed results of the quality assessment tool for cross-sectional and prospective clinical studies.

| **Cross-sectional studies** | **1. Were the criteria for inclusion in the sample clearly defined?** | **2. Were the study subjects and the setting described in detail?** | **3. Was the exposure measured in a valid and reliable way?** | **4. Were objective, standard criteria used for measurement of the condition?** | **5. Were confounding factors identified?** | **6. Were strategies to deal with confounding factors stated?** | **7. Were the outcomes measured in a valid and reliable way?** | **8. Was appropriate statistical analysis used?** | **Positive responses** | **Quality score** |  |  |  |
| --- | --- | --- | --- | --- | --- | --- | --- | --- | --- | --- | --- | --- | --- |
| Ahlgren (1967) | 0 | 2 | 0 | 1 | 1 | 1 | 0 | 0 | 3 | 37,5 |  |  |  |
| Ahlgren et al. (1973) | 0 | 2 | 2 | 2 | 2 | 0 | 2 | 2 | 0 | 0 |  |  |  |
| Alarcon et al. (2000) | 1 | 2 | 2 | 1 | 2 | 2 | 2 | 2 | 2 | 25 |  |  |  |
| Andrade et al. (2010) | 1 | 2 | 0 | 1 | 0 | 0 | 2 | 1 | 3 | 37,5 |  |  |  |
| Andrade et al. (2009) | 1 | 0 | 1 | 1 | 0 | 0 | 1 | 1 | 5 | 62,5 |  |  |  |
| Ben-Bassat et al. (1993) | 0 | 2 | 0 | 1 | 2 | 0 | 1 | 1 | 3 | 37,5 |  |  |  |
| Castelo et al. (2007) | 1 | 2 | 2 | 1 | 1 | 1 | 1 | 1 | 6 | 75 |  |  |  |
| Castelo et al. (2010) | 1 | 2 | 1 | 1 | 1 | 1 | 1 | 1 | 7 | 87,5 |  |  |  |
| Ciavarella et al. (2012) | 1 | 0 | 1 | 1 | 0 | 0 | 1 | 2 | 4 | 50 |  |  |  |
| Ciccone et al. (2010) | 1 | 2 | 0 | 1 | 0 | 0 | 1 | 1 | 4 | 50 |  |  |  |
| Corrêa et al. (2018) | 1 | 2 | 0 | 1 | 0 | 0 | 2 | 2 | 2 | 25 |  |  |  |
| Costa (2020) | 1 | 0 | 1 | 2 | 0 | 0 | 1 | 1 | 4 | 50 |  |  |  |
| Ferrario et al. (1999) | 1 | 2 | 1 | 1 | 1 | 1 | 1 | 2 | 6 | 75 |  |  |  |
| Gaviao et al. (2001) | 1 | 2 | 1 | 1 | 1 | 1 | 2 | 1 | 6 | 75 |  |  |  |
| Henrikson et al. (1998) | 2 | 0 | 1 | 1 | 1 | 1 | 1 | 1 | 6 | 75 |  |  |  |
| Hinotume et al. (1994) | 1 | 0 | 2 | 1 | 0 | 0 | 2 | 2 | 2 | 25 |  |  |  |
| Lenguas et al. (2012) | 1 | 2 | 1 | 1 | 0 | 0 | 1 | 1 | 5 | 62,5 |  |  |  |
| Lowe and Takada (1984) | 1 | 0 | 1 | 1 | 0 | 0 | 1 | 2 | 4 | 50 |  |  |  |
| Nagata et al. (2002) | 2 | 0 | 2 | 0 | 0 | 0 | 1 | 2 | 1 | 12,5 |  |  |  |
| Piancino et al. 2012 | 1 | 1 | 2 | 1 | 0 | 0 | 1 | 1 | 5 | 62,5 |  |  |  |
| Piancino, Isola et al. 2012 | 1 | 1 | 1 | 1 | 1 | 1 | 1 | 1 | 8 | 100 |  |  |  |
| Proffit and Fields (1983) | 1 | 2 | 2 | 1 | 2 | 2 | 1 | 1 | 4 | 50 |  |  |  |
| Regalo et al. (2018) | 1 | 2 | 1 | 1 | 2 | 2 | 1 | 2 | 4 | 50 |  |  |  |
| Rentes et al. (2002) | 1 | 2 | 1 | 2 | 1 | 1 | 1 | 1 | 6 | 75 |  |  |  |
| Sabashi et al. (2009) | 1 | 1 | 1 | 2 | 1 | 1 | 1 | 1 | 7 | 87,5 |  |  |  |
| Salioni et al. (2005) | 1 | 1 | 2 | 1 | 2 | 0 | 1 | 1 | 5 | 62,5 |  |  |  |
| Sever et al. (2011) | 1 | 2 | 0 | 1 | 1 | 0 | 1 | 1 | 5 | 62,5 |  |  |  |
| Shiere and Manly (1952) | 0 | 2 | 2 | 0 | 0 | 0 | 0 | 0 | 0 | 0 |  |  |  |
| Sonnesen et al. (2001) | 1 | 2 | 1 | 1 | 1 | 1 | 1 | 1 | 7 | 87,5 |  |  |  |
| Takada and Lowe (1985) | 0 | 0 | 1 | 1 | 0 | 0 | 1 | 0 | 3 | 37,5 |  |  |  |
| Toro et al. (2006) | 1 | 1 | 1 | 1 | 1 | 1 | 1 | 1 | 8 | 100 |  |  |  |
| Yousefzadeh et al. (2010) | 2 | 2 | 1 | 1 | 1 | 1 | 1 | 1 | 6 | 75 |  |  |  |
| **Prospective studies** | **1. Were the two groups similar and recruited from the same population?** | **2. Were the exposures measured similarly to assign people to both exposed and unexposed groups?** | **3. Was the exposure measured in a valid and reliable way?** | **4. Were confounding factors identified?** | **5. Were strategies to deal with confounding factors stated?** | **6. Were the groups/participants free of the outcome at the start of the study (or at the moment of exposure)?** | **7. Were the outcomes measured in a valid and reliable way?** | **8. Was the follow up time reported and sufficient to be long enough for outcomes to occur?** | **9. Was follow up complete, and if not, were the reasons to loss to follow up described and explored?** | **10. Were strategies to address incomplete follow up utilized?** | **11. Was appropriate statistical analysis used?** | **Positive responses** | **Quality score** |
| Alarcon et al. (2009) | 1 | 1 | 1 | 1 | 1 | 3 | 1 | 3 | 3 | 3 | 1 | 7 | 63,64 |
| Antonarakis and Kiliaridis (2015) | 1 | 1 | 2 | 1 | 1 | 1 | 1 | 1 | 1 | 1 | 1 | 10 | 90,91 |
| Atonarakis et al. (2012) | NA | NA | 1 | 1 | 1 | 1 | 1 | 1 | 2 | 2 | 1 | 7 | 77,78 |
| Atonarakis et al. (2013) | NA | NA | 1 | 1 | 1 | 1 | 1 | 1 | 2 | 2 | 1 | 7 | 77,78 |
| Barrera et al. (2011) | 1 | 1 | 1 | 1 | 1 | 1 | 1 | 1 | 1 | 1 | 1 | 11 | 100 |
| Di Palma et al. (2017) | 2 | 1 | 1 | 0 | 2 | 2 | 1 | 1 | 2 | 2 | 1 | 5 | 45,45 |
| Galbiati et al. (2016) | 1 | 1 | 1 | 2 | 0 | 1 | 2 | 1 | 2 | 0 | 0 | 5 | 45,45 |
| Go (1981) | 0 | 0 | 0 | 2 | 0 | 1 | 1 | 1 | 2 | 0 | 2 | 3 | 27,27 |
| Henrikson et al. (2009) | 1 | 1 | 2 | 0 | 0 | 1 | 1 | 1 | 1 | 0 | 2 | 6 | 54,55 |
| Ingervall and Thuer (1991) | 0 | 1 | 1 | 2 | 2 | 2 | 1 | 1 | 2 | 0 | 1 | 5 | 45,45 |
| Kecik et al. (2007) | 1 | 1 | 1 | 0 | 0 | 1 | 1 | 1 | 1 | 2 | 1 | 8 | 72,73 |
| Keeling et al. (1991) | 0 | 1 | 1 | 0 | 0 | 2 | 2 | 2 | 2 | 2 | 1 | 3 | 27,27 |
| Martin et al. (2012) | 1 | 1 | 2 | 1 | 2 | 1 | 1 | 1 | 2 | 0 | 1 | 7 | 63,64 |
| Michelotti et al. (2019) | 1 | 1 | 1 | 1 | 2 | 1 | 1 | 1 | 2 | 0 | 1 | 8 | 72,73 |
| Nuno-Licona et al. (1993) | 2 | 1 | 1 | 2 | 0 | 0 | 1 | 2 | 2 | 0 | 2 | 3 | 27,27 |
| Petrovic et al. (2014) | 2 | 1 | 1 | 0 | 0 | 2 | 1 | 1 | 2 | 0 | 1 | 5 | 45,45 |
| Piancino et al. (2016) | 1 | 1 | 2 | 1 | 1 | 1 | 1 | 1 | 2 | 2 | 1 | 8 | 72,73 |
| Piancino et al. (2017) | 1 | 1 | 2 | 0 | 2 | 1 | 1 | 1 | 2 | 0 | 1 | 6 | 54,55 |
| Roldan et al. (2016) | 1 | 1 | 2 | 1 | 1 | 1 | 1 | 1 | 1 | 2 | 1 | 9 | 81,82 |
| Satygo et al. (2014) | 1 | 1 | 1 | 0 | 2 | 1 | 1 | 1 | 2 | 0 | 1 | 7 | 63,64 |
| Spolaor et al. (2020) | 2 | 1 | 1 | 0 | 2 | 0 | 1 | 1 | 1 | 1 | 1 | 7 | 63,64 |
| Throckmorton et al. (2001) | 1 | 1 | 2 | 1 | 1 | 2 | 1 | 1 | 2 | 0 | 1 | 7 | 63,64 |
| Yashiro et al. (2004) | 2 | 1 | 0 | 2 | 2 | 1 | 1 | 1 | 2 | 0 | 1 | 5 | 45,45 |
| **Abbreviations:** NA; not applicable, 0; No, 1; Yes, 2; Unclear Quality score: Below 60 = Low, 60-79 = Moderate, and 80-100 = High | | | | | | | | | | | | | |
