# Supplementary material for: Effect of malocclusion on jaw motor function and chewing in children: a systematic review
Source: Clin Oral Investig. 2022 Jan 5;26(3):2335–51. doi: 10.1007/s00784-021-04356-y (PMC8898242; doi:10.1007/s00784-021-04356-y)
Supplement: Supplementary file 3 — Supplementary file3 (DOC 55 KB) [file 784_2021_4356_MOESM3_ESM.doc]

**Summary of findings:**

# The influence of orthodontic treatment on MOBF in children with malocclusion

**Patient or population**: children **Setting**:

**Intervention**: Orthodontic treatment **Comparison**:

| Outcomes | Impact | | № of participants  (studies) | | Certainty of the evidence (GRADE) | |
| --- | --- | --- | --- | --- | --- | --- |
| Orthodontic treatment effect of MOBF in children with Cl II/1 malocclusion | | The MOBF in children with Class II/1 malocclusion increased during 1 to 2 years before orthodontic functional treatment but decreased during treatment. After one year of retention, the MOBF in children with Class II/1 increased to reach their pre-functional orthodontic treatment bite force levels. | | 93  (3 observational studies) ^1–3^ | | ⨁⨁⨁◯  Moderate ^a^ |

***The risk in the intervention group** (and its 95% confidence interval) is based on the assumed risk in the comparison group and the **relative effect** of the intervention (and its 95% CI).

**CI:** Confidence interval

**GRADE Working Group grades of evidence**

**High certainty:** We are very confident that the true effect lies close to that of the estimate of the effect

**Moderate certainty:** We are moderately confident in the effect estimate: The true effect is likely to be close to the estimate of the effect, but there is a possibility that it is substantially different

**Low certainty:** Our confidence in the effect estimate is limited: The true effect may be substantially different from the estimate of the effect

**Very low certainty:** We have very little confidence in the effect estimate: The true effect is likely to be substantially different from the estimate of effect

**Explanations**

1. No control group in two studies

**References**

1. Antonarakis GS, Kjellberg H, Kiliaridis S. Bite force and its association with stability following Class II/1 functional appliance treatment. Eur J Orthod [Internet]. 2013;35(4):434–41.

2. Antonarakis GS, Kjellberg H, Kiliaridis S. Predictive value of molar bite force on Class II functional appliance treatment outcomes. Eur J Orthod [Internet]. 2012;34(2):244–9.

3. Antonarakis GS, Kiliaridis S. Predictive value of masseter muscle thickness and bite force on Class II functional appliance treatment: a prospective controlled study. Eur J Orthod [Internet]. 2015;37(6):570–7.
