# Supplementary material for: Effect of malocclusion on jaw motor function and chewing in children: a systematic review
Source: Clin Oral Investig. 2022 Jan 5;26(3):2335–51. doi: 10.1007/s00784-021-04356-y (PMC8898242; doi:10.1007/s00784-021-04356-y)
Supplement: Supplementary file 4 — Supplementary file4 (DOC 56 KB) [file 784_2021_4356_MOESM4_ESM.doc]

**Summary of findings:**

# The influence of orthodontic treatment on EMG activity in children with malocclusion

**Patient or population**: children **Setting**:

**Intervention**: Orthodontic treatment **Comparison**:

| Outcomes | Impact | | № of participants  (studies) | | Certainty of the evidence (GRADE) | |
| --- | --- | --- | --- | --- | --- | --- |
| Orthodontic treatment effect on EMG activity in children with UPXB | | The EMG activity of masseter and/or anterior temporalis muscles in children with UPXB improved after orthodontic treatment and retention reaching normal levels. | | 384  (6 observational studies) ^1–6^ | | ⨁⨁⨁◯  MODERATE a |
| Orthodontic treatment effect on EMG activity in children with Cl I, II and III malocclusions | | Three studies found that EMG activities of masseter and temporalis muscles in children with Class I, Class II/1 or Class III malocclusions were stable, regardless of treatment type. Another three studies found that functional orthodontic treatment of children with Class II/1 and/or II/2 malocclusions resulted in an increase ^7,8^ or decrease in the masseter and temporalis muscle EMG activity during mandibular clenching, which reached control levels after treatment. | | 51 (6 observational studies) ^7–11^ | | ⨁◯◯◯  VERY LOW b,c,d |

***The risk in the intervention group** (and its 95% confidence interval) is based on the assumed risk in the comparison group and the **relative effect** of the intervention (and its 95% CI).

**CI:** Confidence interval

**GRADE Working Group grades of evidence**

**High certainty:** We are very confident that the true effect lies close to that of the estimate of the effect

**Moderate certainty:** We are moderately confident in the effect estimate: The true effect is likely to be close to the estimate of the effect, but there is a possibility that it is substantially different

**Low certainty:** Our confidence in the effect estimate is limited: The true effect may be substantially different from the estimate of the effect

**Very low certainty:** We have very little confidence in the effect estimate: The true effect is likely to be substantially different from the estimate of effect

**Explanations**

1. No control group in one study
2. No control group in two studies
3. Confounders were not identified or dealt with in all the studies
4. different samples and subgroups between studies, leading to heterogeneity in results
5. Indirectness due to substantial differences between the population, the intervention, or the outcomes measured in relevant research studies e. variability in sample age and gender was not equally distributed among subgroups

**References**

1. Piancino MG, Falla D, Merlo A, Vallelonga T, de Biase C, Dalessandri D, et al. Effects of therapy on masseter activity and chewing kinematics in patients with unilateral posterior crossbite. Arch Oral Biol [Internet]. 2016 Jul;67:61–7.

2. Spolaor F, Mason M, De Stefani A, Bruno G, Surace O, Guiotto A, et al. Effects of Rapid Palatal Expansion on Chewing Biomechanics in Children with Malocclusion: A Surface Electromyography Study. Sensors (Basel). 2020 Apr 7;20(7):2086.

3. Michelotti A, Rongo R, Valentino R, D’Antò V, Bucci R, Danzi G, et al. Evaluation of masticatory muscle activity in patients with unilateral posterior crossbite before and after rapid maxillary expansion. Eur J Orthod. 2019 Jan 23;41(1):46–53.

4. Galbiati G, Maspero C, Giannini L, Tagliatesta C, Farronato G. Functional evaluation in young patients undergoing orthopedical interceptive treatment. Minerva Stomatol [Internet]. 2016 Oct 23;65(5):276–83.

5. Kecik D, Kocadereli I, Saatci I. Evaluation of the treatment changes of functional posterior crossbite in the mixed dentition. Am J Orthod Dentofac Orthop. 2007 Feb;131(2):202–15.

6. Martín C, Palma JC, Alamán JM, Lopez-Quiñones JM, Alarcón JA. Longitudinal evaluation of sEMG of masticatory muscles and kinematics of mandible changes in children treated for unilateral cross-bite. J Electromyogr Kinesiol. 2012;22(4):620–8.

7. Satygo EA, Silin A V, Ramirez-Yañez GO. Electromyographic muscular activity improvement in Class II patients treated with the pre-orthodontic trainer. J Clin Pediatr Dent [Internet]. 2014;38(4):380–4.

8. Petrović D, Vujkov S, Petronijević B, Šarčev I, Stojanac I. Examination of the bioelectrical activity of the masticatory muscles during Angle’s Class II division 2 therapy with an activator. Vojnosanit Pregl [Internet]. 2014 Dec;71(12):1116–22.

9. Di Palma E, Tepedino M, Chimenti C, Tartaglia GM, Sforza C. Effects of the functional orthopaedic therapy on masticatory muscles activity. J Clin Exp Dent [Internet]. 2017 Jul;9(7):e886–91.

10. Nuño-Licona A, Cavazos Jr E, Angeles-Medina F. Electromyographic changes resulting from orthodontic correction of class III malocclusion. Int J Paediatr Dent. 1993;3(2):71–6.

11. Ingervall B, Thüer U. Temporal muscle activity during the first year of Class II, division 1 malocclusion treatment with an activator. Am J Orthod Dentofacial Orthop [Internet]. 1991 Apr;99(4):361–8.
