# Supplementary material for: Effect of malocclusion on jaw motor function and chewing in children: a systematic review
Source: Clin Oral Investig. 2022 Jan 5;26(3):2335–51. doi: 10.1007/s00784-021-04356-y (PMC8898242; doi:10.1007/s00784-021-04356-y)
Supplement: Supplementary file 5 — Supplementary file5 (DOC 54 KB) [file 784_2021_4356_MOESM5_ESM.doc]

**Summary of findings:**

# The influence of orthodontic treatment on jaw kinematics in children with malocclusion

**Patient or population**: children **Setting**:

**Intervention**: Orthodontic treatment **Comparison**:

| Outcomes | Impact | | № of participants  (studies) | | Certainty of the evidence (GRADE) | |
| --- | --- | --- | --- | --- | --- | --- |
| Orthodontic treatment effect on jaw kinematics in children with UPXB | | Orthodontic treatment of children with UPXB resolved the wider jaw closing angle and significantly decreased the reverse chewing cycles while chewing. | | 293  (5 observational studies) ^1–5^ | | ⨁⨁⨁◯  MODERATE a |
| Orthodontic treatment effect on jaw kinematics in children with AXB | | The variability of jaw chewing pattern decreased after orthodontic treatment in children with anterior crossbite and became similar to controls. | | 21  (1 observational study) ^6^ | | ⨁⨁◯◯  LOW b,c |

***The risk in the intervention group** (and its 95% confidence interval) is based on the assumed risk in the comparison group and the **relative effect** of the intervention (and its 95% CI).

**CI:** Confidence interval

**GRADE Working Group grades of evidence**

**High certainty:** We are very confident that the true effect lies close to that of the estimate of the effect

**Moderate certainty:** We are moderately confident in the effect estimate: The true effect is likely to be close to the estimate of the effect, but there is a possibility that it is substantially different

**Low certainty:** Our confidence in the effect estimate is limited: The true effect may be substantially different from the estimate of the effect

**Very low certainty:** We have very little confidence in the effect estimate: The true effect is likely to be substantially different from the estimate of effect

**Explanations**

1. The presence of mandibular functional shift was not clarified in 3 studies
2. Exposure was not similar in the intervention group
3. Confounding factors where not identified or dealt with

**References**

1. Piancino MG, Falla D, Merlo A, Vallelonga T, de Biase C, Dalessandri D, et al. Effects of therapy on masseter activity and chewing kinematics in patients with unilateral posterior crossbite. Arch Oral Biol [Internet]. 2016 Jul;67:61–7.

2. Piancino MG, Cordero-Ricardo M, Cannavale R, Vallelonga T, Garagiola U, Merlo A. Improvement of masticatory kinematic parameters after correction of unilateral posterior crossbite: Reasons for functional retention. Angle Orthod. 2017;87(6):871–7.

3. Throckmorton GS, Buschang PH, Hayasaki H, Pinto AS. Changes in the masticatory cycle following treatment of posterior unilateral crossbite in children. Am J Orthod Dentofac Orthop [Internet]. 2001;120(5):521–9.

4. Ben-Bassat Y, Yaffe A, Brin I, Freeman J, Ehrlich Y. Functional and morphological-occlusal aspects in children treated for unilateral posterior cross-bite. Eur J Orthod [Internet]. 1993;15(1):57–63.

5. Martín C, Palma JC, Alamán JM, Lopez-Quiñones JM, Alarcón JA. Longitudinal evaluation of sEMG of masticatory muscles and kinematics of mandible changes in children treated for unilateral cross-bite. J Electromyogr Kinesiol. 2012;22(4):620–8.

6. Yashiro K, Miyawaki S, Takada K. Stabilization of jaw-closing movements during chewing after correction of incisor crossbite. J Oral Rehabil [Internet]. 2004;31(10):949–56.
