# Supplementary material for: Effect of malocclusion on jaw motor function and chewing in children: a systematic review
Source: Clin Oral Investig. 2022 Jan 5;26(3):2335–51. doi: 10.1007/s00784-021-04356-y (PMC8898242; doi:10.1007/s00784-021-04356-y)
Supplement: Supplementary file 6 — Supplementary file6 (DOC 54 KB) [file 784_2021_4356_MOESM6_ESM.doc]

**Summary of findings:**

# The influence of orthodontic treatment on chewing efficiency in children with malocclusion

**Patient or population**: children **Setting**:

**Intervention**: Orthodontic treatment **Comparison**:

| Outcomes | Impact | | № of participants  (studies) | | Certainty of the evidence (GRADE) | |
| --- | --- | --- | --- | --- | --- | --- |
| Orthodontic treatment effect on chewing efficiency in children with Cl II malocclusion | | Chewing efficiency in girls with orthodontically corrected Class II malocclusion was similar to girls with untreated Class II malocclusion but remains reduced compared to controls. | | 183  (1 observational study) ^1^ | | ⨁⨁◯◯  LOW ^a,b^ |

***The risk in the intervention group** (and its 95% confidence interval) is based on the assumed risk in the comparison group and the **relative effect** of the intervention (and its 95% CI).

**CI:** Confidence interval

**GRADE Working Group grades of evidence**

**High certainty:** We are very confident that the true effect lies close to that of the estimate of the effect

**Moderate certainty:** We are moderately confident in the effect estimate: The true effect is likely to be close to the estimate of the effect, but there is a possibility that it is substantially different

**Low certainty:** Our confidence in the effect estimate is limited: The true effect may be substantially different from the estimate of the effect

**Very low certainty:** We have very little confidence in the effect estimate: The true effect is likely to be substantially different from the estimate of effect

**Explanations**

1. Generalizability issues due to recruitment of girls only
2. Confounders where not identified or dealt with

**References**

1. Henrikson T, Ekberg E, Nilner M. Can orthodontic treatment improve mastication? A controlled, prospective and longitudinal study. Swed Dent J [Internet]. 2009;33(2):59–65.
